# PAX5 is part of a functional transcription factor network targeted in lymphoid leukemia

**DOI:** 10.1371/journal.pgen.1008280

**Published:** 2019-08-05

**Authors:** Kazuki Okuyama, Tobias Strid, Jacob Kuruvilla, Rajesh Somasundaram, Susana Cristobal, Emma Smith, Mahadesh Prasad, Thoas Fioretos, Henrik Lilljebjörn, Shamit Soneji, Stefan Lang, Jonas Ungerbäck, Mikael Sigvardsson

**Affiliations:** 1 Department of Clinical and Experimental Medicine, Linköping University, Linköping, Sweden; 2 Division of Molecular Hematology, Lund University, Lund, Sweden; 3 Division of Clinical Genetics Lund University, Lund, Sweden; 4 Lund Stemcell Center, Lund University, Lund, Sweden; St. Jude Children's Research Hospital, UNITED STATES

## Abstract

One of the most frequently mutated proteins in human B-lineage leukemia is the transcription factor PAX5. These mutations often result in partial rather than complete loss of function of the transcription factor. While the functional dose of PAX5 has a clear connection to human malignancy, there is limited evidence for that heterozygote loss of PAX5 have a dramatic effect on the development and function of B-cell progenitors. One possible explanation comes from the finding that PAX5 mutated B-ALL often display complex karyotypes and additional mutations. Thus, PAX5 might be one component of a larger transcription factor network targeted in B-ALL. To investigate the functional network associated with PAX5 we used BioID technology to isolate proteins associated with this transcription factor in the living cell. This identified 239 proteins out of which several could be found mutated in human B-ALL. Most prominently we identified the commonly mutated IKZF1 and RUNX1, involved in the formation of ETV6-AML1 fusion protein, among the interaction partners. ChIP- as well as PLAC-seq analysis supported the idea that these factors share a multitude of target genes in human B-ALL cells. Gene expression analysis of mouse models and primary human leukemia suggested that reduced function of PAX5 increased the ability of an oncogenic form of IKZF1 or ETV6-AML to modulate gene expression. Our data reveals that PAX5 belong to a regulatory network frequently targeted by multiple mutations in B-ALL shedding light on the molecular interplay in leukemia cells.

## Author summary

The use of modern high throughput DNA-sequencing has dramatically increased our ability to identify genetic alterations associated with cancer. However, while the mutations per se are rather easily identified, our understanding of how these mutations impact cellular functions and drive malignant transformation is more limited. We have explored the function of the transcription factor PAX5, commonly mutated in human B-lymphocyte leukemia, to identify a regulatory network of transcription factors often targeted in human disease. Hence, we propose that malignant conversion of B-lymphocyte progenitors involves multiple targeting of a central transcription factor network aggravating the impact of the individual mutations. These data increase our understanding for how individual mutations collaborate to drive the formation of B—lineage leukemia.

## Introduction

It is becoming increasingly clear that transcription factors essential for normal B-cell development are frequently mutated in B-lineage Acute Lymphoblastic Leukemia (B-ALL) [1]. One of the most common targets for genetic alterations in B-ALL is the transcription factor PAX5 targeted both by translocations generating fusion proteins [2, 3] and by partial inactivation by deletions [4–6] or point mutations [6, 7]. Mutations reducing PAX5 function is one of the most common alterations detected in B-ALL involving about one third of the malignancies [4, 5]. However, the findings that *PAX5* deletion in human B-ALL is associated with complex karyotypes [4, 5] and that reduced function of PAX5 in mouse models generate a mild phenotype [8–10] indicate that PAX5 mutations collaborate with other oncogenic events to cause malignant transformation. This has been verified in mouse models where heterozygote deletion of *Pax5* in combination with expression of constitutively active STAT5 [11] or partial inactivation of the *Ebf1* gene [10] largely increase leukemia formation.

**Fig 1:**
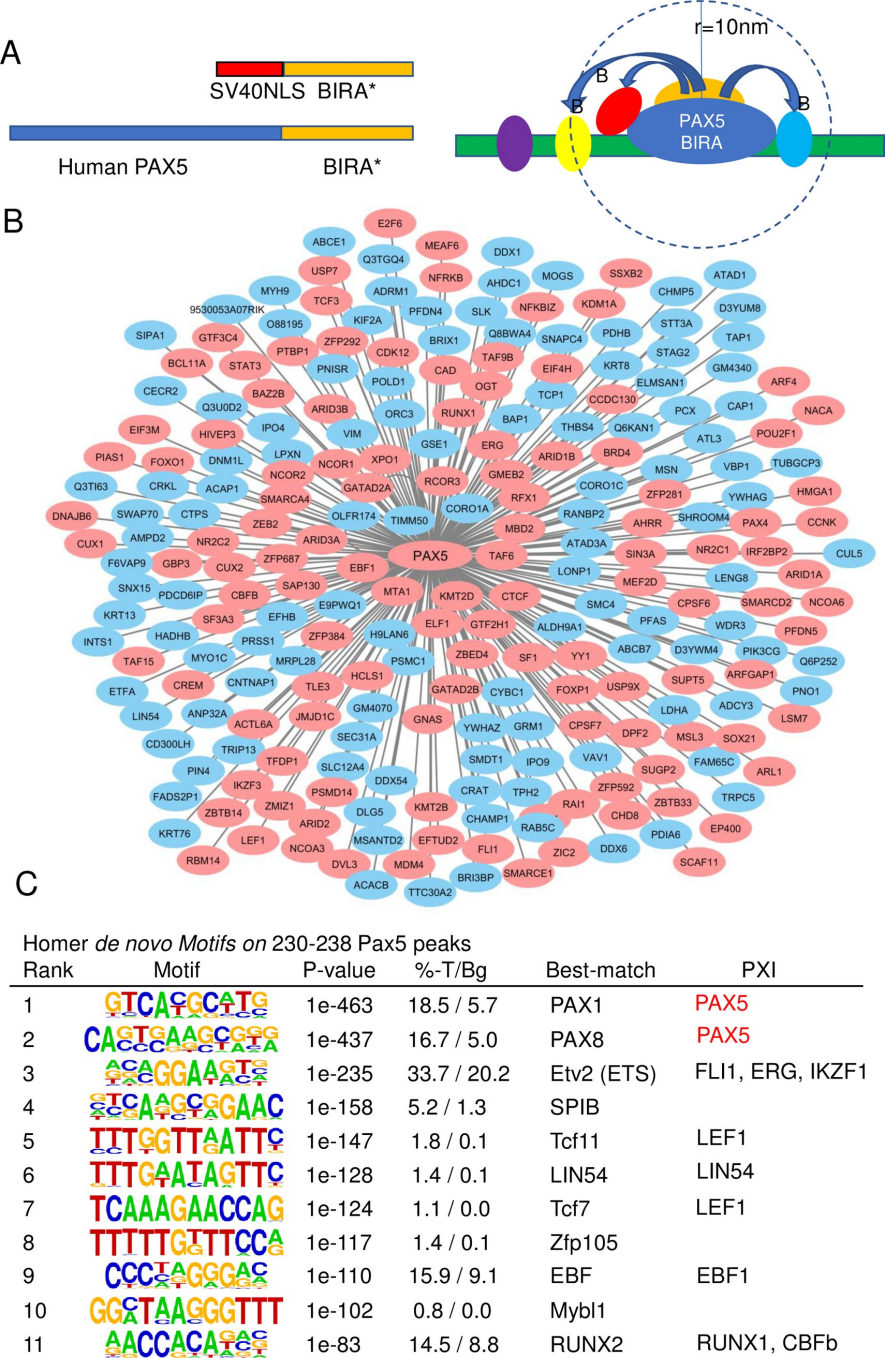
PAX5 is part of a complex regulatory network of DNA binding proteins and co-regulators of transcription. Panel **(A)** display a schematic drawing of the basic principle for PXI identification by BioID and the fusion proteins (PAX5-BIRA* and SV40NLS-BIRA*) used to resolve the interactome of PAX5 in mouse Pre-B cells. (**B**) Cytoscape (version 3.6.1) generated protein interaction map for PAX5 based on BioID data analyzed using the Trans-Proteomic Pipeline (TPP) software and SAINT Express v.3.3. A Bayesian FDR of 0.02 (corresponding to a SAINT score of ~0.80) was used as a cut-off to identify high confidence interactors. The red color of the node indicates a protein defined as involved in transcriptional regulation. The analysis is based on 3 biological and 2 technical replicates with BIRA* conjugated to NLS collapsed to the 2 highest spectral counts for each prey. (**C**) Data from *de novo* motif enrichment analysis (mm10 –*size* 200) for PAX5 sites in mouse 230238 Pre-B cells. T/BG% indicates the frequency of targets enriched for the motif/the background frequency of the motif. Identified PXIs associated with enriched motifs are indicated.

In order to better understand the regulatory networks in B-ALL and to resolve how other genetic events may be functionally linked to *PAX5* mutations we have used BioID to identify collaboration partners for PAX5. BioID is based on the generation of a fusion between the factor of interest and a mutated form of the bacterial protein biotinylase BIRA (BIRA*) [12, 13]. This mutant enzyme lack substrate specificity and covalently attach biotin to any protein within about ~10nm distance of the fusion protein (Fig 1A). Biotinylated proteins are isolated and identified by MS/MS allowing us to identify Proximity Interaction Partners (PXIs) for PAX5 in the living cell. Analysis of the Cosmic data base (http://cancer.sanger.ac.uk/cosmic) [14] revealed that a substantial fraction of the *PAX5* mutated leukemias carried additional mutations in identified PXIs. Among these PXIs were, RUNX1, involved in the 12;21 translocation generating the ETV6-RUNX1 fusion protein, and IKZF1 both commonly mutated together with PAX5 in B-ALL [4, 5]. Using Chromatin Immuno-precipitation (ChIP)-seq, proximity ligation assisted ChIP-seq (PLAC-seq) [15] and RNA-seq analysis we confirmed that PAX5, RUNX1 and IKZF1 share a large number of target genes and that a dominant negative form of IKZF1 or the ETV6-RUNX1 fusion protein acted collaboratively with heterozygote deletion of *Pax5* to modulate gene expression. This suggest that the transformation process in B-ALL involve multiple mutations of genes being part of a regulatory network possibly causing an exacerbated effect on the transcriptional programming in the B-cell progenitor.

## Results

### PAX5 associate with regulators of transcription commonly mutated in human leukemia

To identify interaction partners for PAX5, we generated N-terminal fusions of BIRA* with a full length human PAX5 protein. A control protein was created by fusion of BIRA* to a SV40-Nuclear localization signal (Fig 1A). The fusion proteins were ectopically expressed in a mouse Abelson virus transformed Pre-B cell line (230–238), after which biotinylated proteins were purified on streptavidin beads. Tandem mass spectrometry and bioinformatic analysis (see extended Materials and Methods) identified 239 high confidence (Saint score above 0.8) PAX5 PXIs (Fig 1B, S1 Table).

Among the identified PXIs we found six proteins previously identified as direct interaction partners [16] and STRING (https://string-db.org) based interactome analysis identified another 14 PXIs as linked to PAX5 (S1 Fig, S1 Table). Protein analysis through evolutionary relationships (PANTHER) (http://pantherdb.org/geneListAnalysis.do) analysis (S2 Fig), as well as Gene ontology (GO) analysis performed with PANTHER14.0 Database for Annotation (S1 Table), identified a large number of PXIs as sequence specific DNA binding proteins or transcriptional co-factors. Several PXIs constituted components of complexes previously linked to PAX5 including the SWI/SNF activator- [16] as well as the Mi-2/NuRD-complexes [17] and NCOR1 [16].

Because multiple PXIs represented DNA binding transcription factors we analyzed PAX5 ChIP-seq data from 230–238 Pre-B cells by *de novo* motif enrichment analysis (Fig 1C). Among the top 10 enriched motifs two were identified as PAX binding sites. However, an additional six top ranked motifs represented putative binding sites for TFs identified as PXIs providing an independent line of support for that these proteins indeed share regulatory elements with PAX5. Thus, BioID analysis suggests that PAX5 is part of a complex network of transcriptional regulators in early B-cell progenitors.

**Fig 2:**
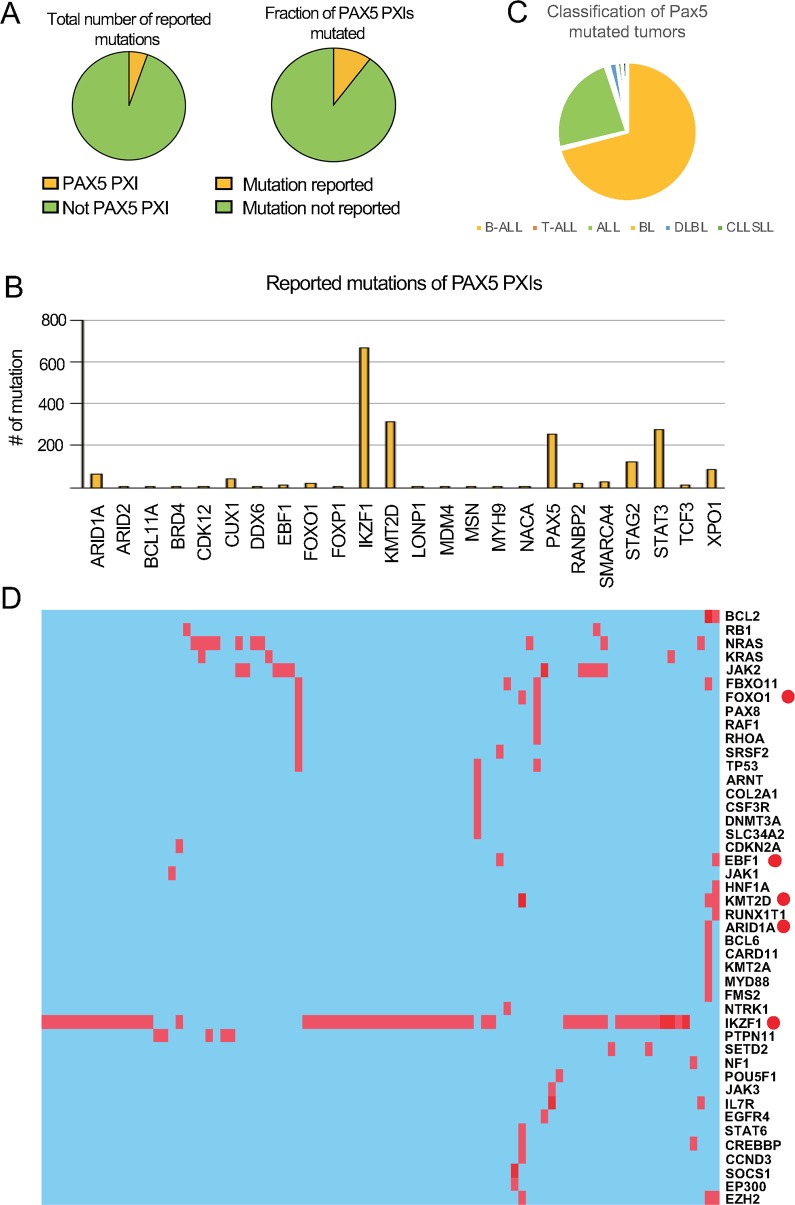
PAX5 PXIs are frequently mutated in combination with PAX5 in human leukemia. (A) Pie charts showing the fraction of PAX5 PXIs (list of 239 entries) found mutated in the public cancer database COSMIC (cancer genes sensus V76) from the Sanger Institute Catalogue Of Somatic Mutations In Cancer web site, (http://cancer.sanger.ac.uk/cosmic)(14). The left chart displays the frequency of mutated PXIs of all reported mutations (a total of 449 genes) in hematological and lymphoid malignancies while the right chart displays the fraction of PAX5 PXIs found mutated in the same malignancies. The diagram in panel (**B**) display the number of *PAX5* and PAX5 PXI mutations identified in hematological malignancies in the database. Panel (**C**) displays a pie-chart presenting the clinical classification of tumors carrying *PAX5* mutations while panel (**D**) displays a heatmap presenting the mutational spectra of 91 unique *PAX5* mutated hematological and lymphoid tumors from the Cosmic database carrying reported co-mutations. A red dot indicates that the mutated gene encode a PAX5 PXI.

To unravel a potential involvement of PAX5 PXIs in human leukemia we used information from the Cosmic cancer mutation database (cancer genes sensus V76) (http://cancer.sanger.ac.uk/cosmic) [14] to estimate the mutation frequency of PAX5 PXIs in human hematopoietic malignancies. Genes encoding PAX5 PXIs covered approximately 7% of the mutations reported (Fig 2A) involving 15% of the PAX associated proteins (Fig 2A). These included IKZF1, ARID1A, KMT2D, STAT3 and PAX5 itself (Fig 2B).

The PXI mutations were enriched in lymphoid and NK lineage malignancies as compared to myeloid leukemias (S3 Fig) indicating a degree of lineage selectivity. To resolve if mutations in PXIs are linked to genetic alterations in *PAX5* we extracted data from *PAX5* mutated hematological malignancies. The absolute majority of these tumors were classified as B-ALL or ALL (Fig 2C). Several of these *PAX5* mutated ALL cells carried additional mutations (Fig 2D). Among these we identified 5 genes encoding identified PAX5 PXIs (Fig 2D). Even though *IKZF1* was the most prominently co-mutated PXI, we did identify tumors carrying combined *PAX5* and *FOXO1*, *EBF1*, *KTM2D* or *ARID1A* mutations (Fig 2D). These data suggest that the transformation process may involve targeting of multiple proteins belonging the same regulatory network.

### PAX5 is part of a functional transcription factor network

**Fig 3:**
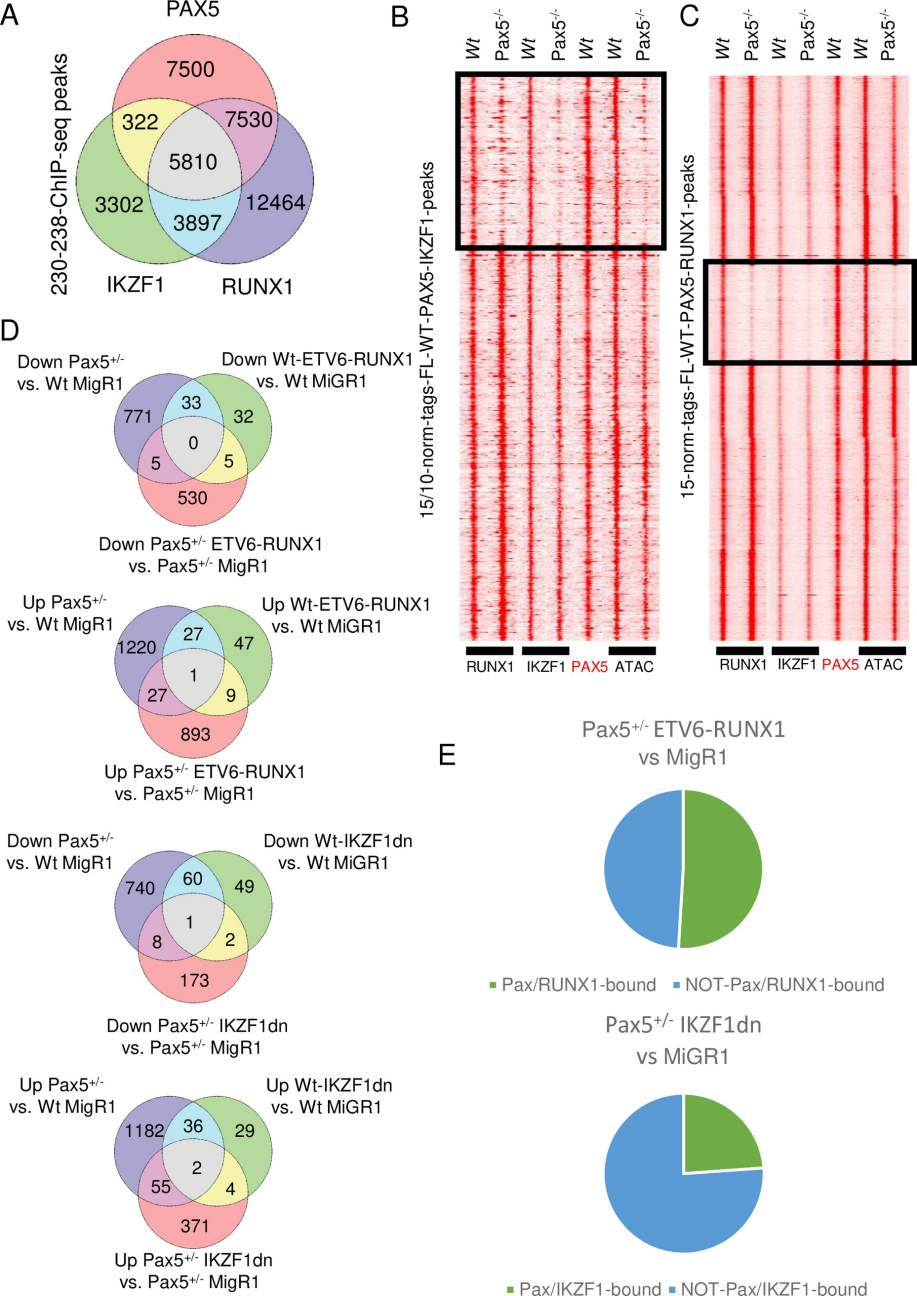
PAX5 is a coordinator of a transcription factor network in pro-B cells. Panel **(A)** display a Venn-diagram of ChIP-seq peaks based on PAX5, IKZF1 and RUNX1 ChIP seq analysis using the 230–238 Pre-B cell line. Peaks were called using the HOMER platform (*findPeaks -style factor*) and resulting files were filtered for peaks ≥15 normalized tags. Overlapping peaks were identified using the *mergePeaks* command in HOMER. **(B-C)** Heat maps displaying overlapping PAX5 and IKZF1 or RUNX1 binding sites as defined by ChIP-seq analysis of *in vitro* expanded *Wt* Pro-B cells. Peaks were identified using HOMER (*findPeaks -style factor*) and filtered for ≥15 (PAX5, RUNX1) or ≥10 (IKZF1) normalized tags. Overlapping peaks were identified using the *mergePeaks* command in HOMER. ChIP-seq and ATAC-seq (GSE92434) (43) signals were visualized in heatmaps with data obtained from primary *in vitro* expanded *Wt* or *Pax5*^*-/-*^ mouse Pre-B cells as indicated. Heatmaps were clustered in Cluster3 using average linkage uncentered correlation. (**D**) Venn diagrams displaying results from RNA-seq experiments from primary *in vitro* expanded FL-*Wt* and *Pax5*^*+/-*^ Pre-B cells transduced with retroviruses encoding either a dominant negative form of IKZF1 (IKZF1-dn), an ETV6-RUNX1 or a control vector (pMIG). Differentially expressed genes (FDR ≤ 0.05, foldchange ≥ 2) were identified using HOMER as described in materials and methods. (**E**) Pie-charts displaying the fraction on genes differentially expressed in ETV6-RUNX1 or IKZF1dn as compared to MIGR1 transduced *Pax*^*+/-*^ cells that can be defined as direct target genes for the transcription factors. Definition of target genes were based on proximity annotation of IKZF1 and RUNX1 peak files merged with PAX5 peaks from 230–238 Pre-B cells to identify IKZF1 and PAX5 or RUNX1 and PAX5 co-bound genes.

While the analysis of the Cosmic data revealed that IKZF1 was the most commonly mutated PXI (Fig 2D), PAX5 mutations are frequently detected in combination with 12:21. translocation [4, 5]. This generates a fusion protein between ETV6 and the DNA binding domain of RUNX1 (ETV6-RUNX1) suggested to act as a repressor at elements normally controlled by RUNX1 [18–20]. To explore the extent to which IKZF1, RUNX1 and PAX5 share regulatory elements we performed ChIP-seq analysis in 230–238 mouse Pre-B cells. This identified just over 21 000 binding sites for PAX5 out of which 65% overlapped with either IKZF1, RUNX1 or both (Fig 3A). Furthermore, 46% of the IKZF1 binding sites and 45% of the RUNX1 sites overlapped with binding of PAX5. Shared binding to target elements in the *Igll1* and *CD79a* promoters could also be verified using ChIP-QPCR (S4A Fig).

*De novo* motif enrichment analysis of peak positions identified enrichment of the expected motif as well as that of putative binding sites for the other factors of this putative network (S4B Fig). In the case of IKZF1 identified as an ETS protein binding site as previously reported [21]. Annotation of shared and unique binding sites to specific targets identified 7046 genes (S2 Table Metadata) that could be assigned to only one of the categories of overlapping or unique binding defined (Fig 3A). GO-term analysis of single, double, or triple bound genes failed to reveal any strong enrichment of genes encoding proteins belonging to any defined biological process (S3 Table). However, genes linked to binding of all three factors were enriched (Benjamini-Hochberg p.adj -value ≤ 0.05) for genes encoding proteins involved in cell cycle and DNA repair as well as protein transport (S3 Table). To explore the expression patterns of the genes targeted by single or multiple binding of PAX5, RUNX1 and/or IKZF1 in the hematopoietic system in mice, we uploaded the unique gene sets defined in S2 Table to Gene Expression commons (https://gexc.riken.jp). As expected *Ikzf1* and *Runx1* were expressed in multiple lineages and stages of development (S5A Fig) while the expression of *Pax5* as well as a set of known PAX5 target genes were restricted to B-lineage cells (S5A and S5B Fig). The expression of genes bound only by IKZF1, was broadly detected in the hematopoietic system (S5B Fig). In contrast expression of genes linked to binding of only RUNX1 was limited and the PAX5 unique gene signature were virtually not expressed in the hematopoietic system (S5B Fig). In sharp contrast we noted high and broad expression of gene sets associated with binding of more than one factor (S5C Fig). These data support the idea that PAX5, IKZF1 and RUNX1 are part of a regulatory network in hematopoiesis.

While *IKZF1* and *RUNX1* are commonly mutated in human malignancies, other PXIs could not be detected as targeted together with PAX5 in our dataset (Fig 2B). These included the Ets-protein FLI1 [22], reported to share binding sites with IKZF1 in B-cell progenitors [21]. In order to explore if PAX5 is part of other regulatory networks not directly targeted in leukemia, we performed ChIP-seq experiments to identify FLI1 binding sites in 230–238 mouse Pre-B cells.

While the majority of the IKZF1 binding sites were shared with FLI1, a defined set of sites were unique for the individual factors (S4D Fig). Annotating the binding sites to genes (S4 Table) followed by GO-term analysis of genes unique to only one binding category (S5 Table) suggested that the genes bound by PAX5, IKZF1 and FLI1 were enriched for functions related to regulation DNA repair and proliferation. The genes annotated to sites unique for combined PAX5-FLI1 binding were broadly expressed (S5D Fig) and coded for proteins involved in DNA-repair (S5 Table). This indicates that PAX5 may be part of several defined transcription factor networks.

The extensive degree of overlapping binding of a lineage and stage specific factor such as PAX5 and broadly expressed proteins such as IKZF1 and RUNX1, expressed earlier in the developmental trajectory (S5A Fig), opens for the possibility that that the early factors act as molecular beacons targeting PAX5 to regulatory elements. In order to explore the interplay between these factors we performed ChIP-seq analysis targeting PAX5, IKZF1 and RUNX1 using *in vitro* expanded primary *Wt* or *Pax5*^*-/-*^ mouse Pre-B cells. While the majority of the shared binding sites were occupied by transcription factors in the absence of PAX5 (Fig 3B and 3C), a finding verified by ChIP-QPCR analyzing binding to the *Igll1* promoter (S4C Fig), a subgroup of sites was not bound by IKZF1 and/or RUNX1 in *Pax5*^*-/- -*^Pre-B cells. Assay for Transposome Accessible Chromatin (ATAC-seq) [23] analysis revealed that sites with reduced IKZF1 or RUNX1 binding in *Pax5*^*-/-*^ cells displayed low epigenetic accessibility as compared to *Wt* cells (Fig 3B and 3C). Hence, PAX5 has the ability to target IKZF1 and RUNX1 to a subset of epigenetically silent regulatory elements.

In order to investigate the functional interplay between PAX5, RUNX1 and IKZF1 we studied the impact of combined heterozygote deletion of *Pax5* in mouse Pre-B cells and expression of ETV6-RUNX1 or a functionally impaired IKZF1-protein (IKZF1DN) lacking all four Zn-fingers in the DNA binding domain which is commonly observed in B-ALL [24, 25]. Comparative analysis of gene expression in *Wt* as compared to *Pax5*^*+/-*^ mouse Pre-B cells transduced with control vector (pMIG) identified 809 downregulated genes (Fig 3D, S6 Table). In addition to *Pax5* itself, we detected reduced expression of several genes encoding proteins identified as PAX5 PXIs. These included transcription factors such as *Ebf1*, *Lef1*, *Foxo1* as well as *Ikzf3* suggesting PAX5 to be directly involved in the regulation of co-factor expression.

We noted no significant downregulation of other classical PAX5 target genes such as *Cd19* or *Cd79a* indicating that B-cell identity is maintained despite reduced expression of several important transcription factors. 1275 genes were upregulated in *Pax5*^*+/-*^ as compared to *Wt* cells and GO-analysis revealed a significant enrichment of genes associated with “immune system process” including *Tlr1*, *Myd88* and *Icosl* (S7 Table). Ectopic expression of ETV6-RUNX in *Wt* Pre-B cells caused significant reduction in the expression of 70 genes and combined deletion of one allele of *Pax5* and ectopic expression of ETV6-RUNX resulted in the repression of 540 genes. Additionally, we detected upregulation of 84 genes upon expression of ETV6-RUNX in *Wt* mouse Pre-B cells and more than 900 genes in *Pax5*^*+/-*^ cells. GO-analysis identified genes involved in “immune system processes” as enriched among both up- and down-regulated ETV6-RUNX1 responsive genes (S7 Table) possibly reflecting a disruption of the differentiation process.

Ectopic expression of IKZF1DN in *Wt* Pre-B cells resulted in downregulation of 112 genes and increased expression of 71 genes while expression in *Pax5*^*+/-*^ cells reduced the levels of 184 and up-regulated mRNA coding for 332 genes as compared to the control cells (Fig 3D, S6 Table). Even though we were able to identify a few genes such as *Il2ra*, *Ifi30* and *Aim2* associated to the GO-term “immune system processes” as upregulated upon DN-IKZF1 expression, the most striking GO-term enrichments was detected for genes classified as involved in “cell adhesion and cell migration” being downregulated in response to expression of the truncated IKZF1 protein (S7 Table). By proximity annotation of ChIP-seq peaks we detected combined PAX5 and RUNX1 binding at over 50% of the genes modulated by ETV6-RUNX1 expression in *Pax5*^*+/-*^ mouse pre-B cells (Fig 3E). The corresponding figure for IKZF1 was close to 25%. Despite the dramatic changes in gene expression patterns, we were unable to detect any short term (4–5 weeks) malignant expansion of these cells after transplantation. Hence, combined reduction of PAX5 dose and expression of oncogenic variants of RUNX1 or IKZF1 resulted in a synergistic modulation of gene expression in line with the idea that these factors constitute a functional network.

### PAX5, IKZF1, RUNX1 and EBF1 form a genetic network in human B-ALL cells

**Fig 4:**
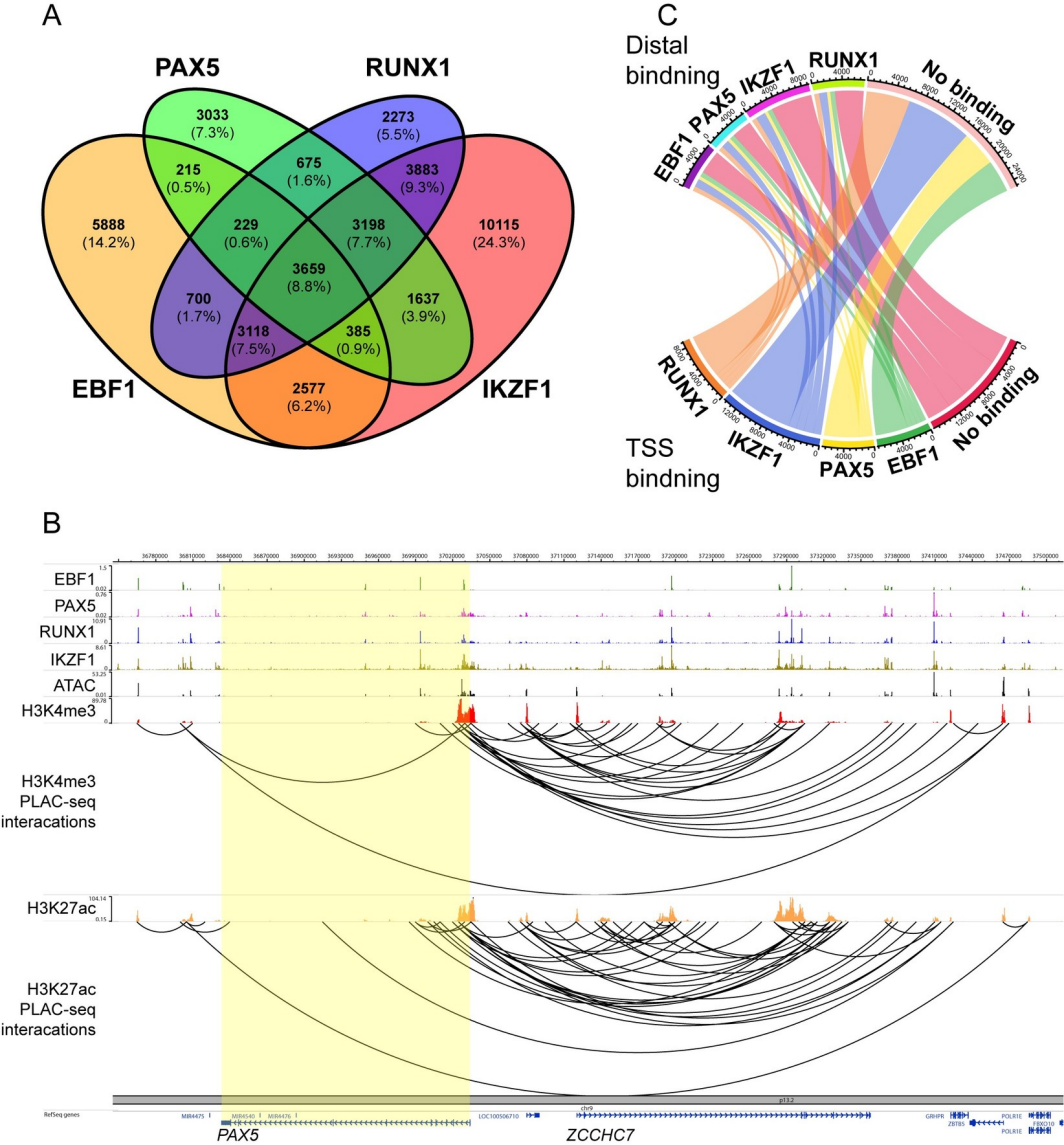
PAX5 is a transcriptional network key regulator in human leukemia cells. Panel **(A**) display a 4-part Venn-diagram based on ChIP-seq experiments targeting PAX5, EBF1, RUNX1 and IKZF1 in the human PreB-ALL cell line NALM6. Overlapping ChIP-seq peaks were identified with the *mergePeaks* command in Homer. (**B**) Visualization of H3K4me3 and H3K27ac anchored PLAC-seq interactions at the *PAX5* gene in NALM6 B-ALL cells. Binding of EBF1, PAX5, RUNX1 and IKZF1 as well as H3K4me3 and H3K27ac chromatin marks was determined by ChIP-seq. ATAC-seq was used to determine chromatin accessibility. The data was displayed using the WashU Genome Browser. (**C**) Chord diagram based on H3K4me3 anchored PLAC-seq from NALM6 displaying interactions between TSS and distal elements. PAX5-EBF1-RUNX1-IKZF1 gene networks anchored distal-to-TSS (transcription start site) are indicated in the diagram. Chromatin loops were filtered for overlapping PAX5, EBF1, RUNX1 or IKZF1 ChIP-seq peaks in either one or both anchor-points (for details, see Methods) with the constraints that one anchor-point had to be within 2.5kb from TSS and the other had to be located more than 2.5kb away from TSS (distal binding). The chord diagram was visualized with the *circlize* package in R.

Having established that PAX5 is part of a functional regulatory network during B-cell development in mice, we wanted to explore the potential collaborative actions of PAX5 and the PXIs IKZF1, RUNX1 as well as EBF1 in human B-ALL cells. To this end we performed ChIP-seq analysis on chromatin from the human B-ALL cell line NALM6 (Fig 4A). We detected overlapping binding of IKZF1 and/or RUNX1 at about 70% of the identified PAX5 binding sites and *de novo* motif enrichment analysis identified binding sites for PAX as well as IKZF1, EBF1 and RUNX1 (S6 Fig). Proximity annotation of TF binding sites revealed that as much as 2645 genes were linked to binding of all four proteins in this human B-ALL cell line.

To explore how the activity of PAX5, IKZF1, RUNX1 and EBF1 are coordinated in the context of the chromatin conformation in human B-ALL cells we performed proximity ligation assisted ChIP-seq (PLAC-seq) analysis [15]. This method combines chromatin capture technology with ChIP-seq to enrich for chromatin interactions associated with binding of a specific protein or a unique protein modification. In order to identify active regulatory elements in NALM6 cells we used ChIP antibodies targeting promoter associated H3K4-trimethylation (H3K4Me3) or H3K27-acetylation (H3K27Ac), marking transcriptionally active elements including enhancers [26, 27]. This allowed us to identify distal interactions (FDR ≤ 0.05; see Methods) between regions separated by at least 10kb in the NALM6 genome with a 5kb resolution.

The complexity of promoter-enhancer interactions is exemplified by analysis of interactions between the human *PAX5* promoter and distal regions (Fig 4B), identifying multiple interactions with elements in the *ZCCHC7* gene. ATAC-seq analysis suggested these distal regions to be epigenetically accessible, and the low level of H3K4Me3 signal in combination with high levels of H3K27Ac (Fig 4B) support the idea that several of these regions represent active enhancer elements [26, 27]. Analysis of ChIP-seq data revealed that multiple regions, including the *PAX5* promoter, are bound by IKZF1, RUNX1 and EBF1 (Fig 4B). Hence, the *PAX5* gene is a target for both autoregulation and potential modulation by RUNX1, IKZF1 and EBF1. Interactions between distal regions at the *EBF1* (S7A Fig), *IKZF1* (S7B Fig) or *RUNX1* genes (S7C Fig) and their promoters identified multiple regions with binding of the transcription factors. This suggests that these four TFs create a regulatory network where these proteins not only share target genes, but also regulate each-others expression via inter- and auto-regulatory loops.

The ChIP-seq analysis provided support for that PAX5, IKZF1, RUNX1 and EBF1 share binding at a substantial set of putative regulatory elements in the human Pre-B cell genome (Fig 4A). In order to investigate the interplay between these TFs over longer distance, we identified interacting regions bound by one or several of the TFs from the PLAC-seq data. Based on H3K27Ac PLAC-seq data, the most common category of interacting elements in the human Pre-B cell genome bound all four TFs at the same region (anchor point (ap)1 or ap2) (S8A Fig). The second most common category contained functional binding sites for all four factors at both the interacting elements. Even though binding of IKZF1 alone to one or the other element was the most commonly detected variant in the DNA-interactome defined by the H3K4Me3 PLAC-seq data (S8B Fig), shared binding of all factors to the same element was prominent also in this data set.

To explore the nature of the long-range interaction in more detail we annotated regions within 2.5kb of a transcriptional start sites (TSS) as promoters. The H3K27Ac PLAC-seq data provides evidence for that the majority of the interactions detected in the human pre-B cell genome were generated through chromatin loops between non-TSS containing elements (S8D Fig). In the H3K4Me3 based data, TSS containing elements were, as expected, enriched and involved in the majority of the detected interactions (Figs 4C and S8C). Binding of all TFs was detected at both promoters and enhancers without any clear specific preference. The major part of the PAX5, IKZF1, RUNX1 or EBF1 bound promoters interacted with enhancers lacking detectable binding of any of these factors. Presented data does, however, support the general idea that PAX5 is part of a regulatory network involving IKZF1 and RUNX1 and EBF1 in human B-ALL cells.

### Mutations of PAX5 and IKZF1 or formation of ETV6-RUNX1 impact target gene expression in primary human leukemia cells

Having identified an extensive functional interplay between PAX5, RUNX1 and IKZF1 we wanted to determine how functional perturbations to this regulatory network impact the expression of direct target genes in primary human B-ALL. In order to link binding of PAX5, EBF1, IKZF1 and RUNX1 to regulatory elements associated with a given gene we used a combinatorial approach of proximity analysis (ChIP-seq) and H3K4Me3-PLAC-seq defined interactions. Because our chromatin configuration analysis allowed for a resolution of 5 kb we assigned TF binding peaks within 2500 base pairs of a TSS as involved in the regulation of the gene defined by the TSS. Elements located at larger distances from the TSS were only assigned to the gene if they were defined as TSS proximal regions in the H3K4Me3 PLAC-assay from NALM6 cells. This approach should increase the precision of the annotation of transcription factor binding sites to defined genes.

**Fig 5:**
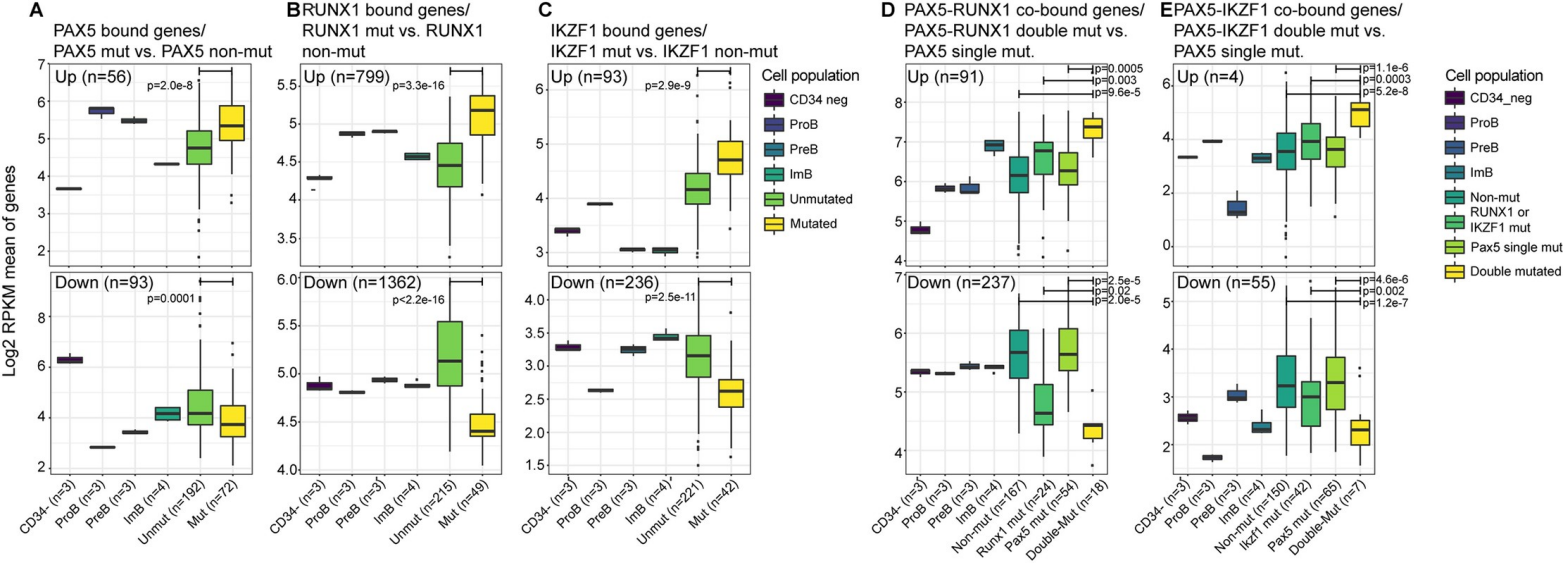
Mutations in B-lineage transcriptional regulators affects expression of their target genes in human leukemia. The figure displays diagrams over a differential expression analysis in a cohort of 264 B-ALL samples and 13 samples from normal HSC (CD34^-^) or B-cell progenitor (Pro-B, Pre-B or Immature B) populations as indicated. PAX5, RUNX1 and/or IKZF1 target genes are defined in the human B-ALL cell line NALM6 using ChIP- and PLAC-sequencing (for details see Methods). Boxplots describe the mean log2 RPKM gene expression for each sample category as defined by differential genes between mutated and unmutated samples **(A-C)**; (**A)** Expression of PAX5 bound genes in *PAX5* mutated vs unmutated leukemia cells, **(B)** RUNX1 bound genes in *RUNX1* mutated vs unmutated or (**C)** IKZF1 bound genes in *IKZF1* mutated vs unmutated B-ALL. Panel **(D, E)** displays boxplots of genes co-bound by (**D)** PAX5-RUNX1 or (**E**) PAX5-IKZF1 differentially expressed in *PAX5* only mutated vs. double mutated tumor cells. n = the number of differential genes between mutated and unmutated samples (A-C) or double-mutated and PAX5 single-mutated samples (D, E). Mann-Whitney *U*-test p-values are shown.

Next, we analyzed the expression of these putative target genes in an RNA-seq data set containing 264 primary B-ALL samples with known mutational status of PAX5 and IKZF1 as well as he presence of ETV6-RUNX1 [6, 28]. To explore the impact of transcription factor mutations on target gene expression we extracted information about mutational status of PAX5, IKZF1 and RUNX1 (ETV6-RUNX1) and classified the tumors as single or double mutants accordingly (S8 Table). Tumors with unknown status for any of these genetic abnormalities were excluded from the analysis. Expression levels of direct transcriptional targets in *PAX5* mutated (*PAX5M*) samples were compared to normal *PAX5* (*PAX5WT*) tumor samples. This identified 56 significantly upregulated and 93 downregulated (p.adj ≤ 0.05) PAX5 target genes (Fig 5A). Considering the large number of binding sites for PAX5 in the genome, only a small fraction of the putative target genes displayed detectable sensitivity to reduced function of PAX5. In order to study if re-expression of PAX5 in a *PAX5* mutant human B-ALL cell would impact the expression of genes identified as downregulated in the patient samples, we took advantage of an RNA-seq dataset generated from REH cells [29]. These cells harbors a mutated PAX5 gene and carry a Tetracyclin inducible PAX5 encoding construct [29] to allow for controlled expression of the factor. 86 genes identified as targeted by PAX5-binding in NALM6 cells and downregulated in *PAX5* mutated primary B-ALL were found to be more PAX5-responsive than PAX5 unbound or bound but not downregulated in primary B-ALL (S8E Fig). This suggests that the approach we have taken allows us to identify relevant PAX5 target genes in primary patient samples. Gene expression analysis of RUNX1 target genes in ETV6-RUNX positive tumors identified 799 up- and 1362 down-regulated genes (Fig 5B) supporting the idea that this fusion protein indeed target RUNX1 regulated genes. IKZF1 mutated cells had a higher expression of 93 and lower expression of 236 direct target genes (Fig 5C) out of the 6138 IKZF1 bound genes expressed in the B-lineage cells.

Hence, while ETV6-RUNX1 expression in primary human leukemia cells associates with altered expression of a substantial fraction of the RUNX1 target genes, reduced function of IKZF1 or PAX5 had limited impact on the expression of their target genes. GO-analysis based on the differentially expressed genes linked to TF mutations revealed little evidence for that this would result in dramatic changes in cellular function. A significant enrichment (Benjamini-Hochberg p.adj ≤ 0.05) was detected for GO-terms linked to cell proliferation and metabolism among the upregulated RUNX1 target genes while the down regulated genes were enriched for GO-terms linked to protein transport and transcription (S9 Table). In general, among the genes identified as differentially expressed within the different categories, a large proportion consisted of transcription factors and epigenetic modulators (S8 and S9 Tables), indicating that disruptions in these transcription factor networks may be part of a general mechanism of action.

To assay whether shared target genes displayed a synergistic impact of combined PAX5 and RUNX1 loss, we used ChIP-seq data and binding site annotation as above to identify 3362 shared target genes expressed in the B-lineage cells. Analyzing the expression levels of these in PAX5 mutated as compared to PAX5M/ETV6-RUNX1 tumors identified 91 up- and 237 down- regulated genes (Fig 5D). Comparing expression of these genes in ETV6-RUNX1 carrying tumors with normal PAX5 genes to double mutant tumors revealed a significant difference in expression supporting the idea that the two targeting events are associated with an exacerbation of the impact on gene expression. Despite that GO-analysis did not detect statistically significant enrichment of any specific GO term describing cellular processes, we noted downregulation of CDKN1a as well as changes in genes involved in the regulation of cell cycle and apoptosis (S9 Table). We were also able to detect similar effects on a low number (4 up and 55 down-regulated genes) of PAX5-IKZF1 targeted cells (Fig 5E). Hence, even though the mutations in this regulatory network impact the expression of a subset of target genes, it does not cause a collapse of stage and lineage specific programs. A finding well in line with the stable cellular state of pre-B-ALL cells.

## Discussion

PAX5 as a frequent target for mutations in B-ALL indicates a central role in the malignant conversion of B-lineage cells [3–7, 30]. Considering its role in normal B-cell differentiation in mice [8] it has been suggested that functional impairment of PAX5 cause a developmental arrest. This idea is supported by the findings that mutant forms of human PAX5 are functionally impaired [6, 7] and that restoration of *PAX5* expression in a human leukemia cell allows for a progressive maturation of the B-ALL like cells and loss of malignant phenotype [29]. However, a complete block of differentiation as imposed by RAG deficiency, do not result in leukemia formation in collaboration with activated STAT5 as efficiently as heterozygote loss of *Pax5 in* mouse models [11]. Furthermore, it has been reported that PAX5 acts as a metabolic gatekeeper so that partial inactivation results in increased metabolic activity in human B-cell progenitors, likely promoting a malignant state [31]. In addition, PAX5 act as a critical regulator of cell identity and reduced function results in lineage plasticity in normal as well as malignant B-lineage cells from both mouse and humans [32–34]. These findings highlight the complex function of this transcription factor in normal and malignant B-cell development and expose our limited understanding of the mechanism of action in the leukemogenesis process likely extending far beyond impaired differentiation.

Despite that the *PAX5* gene is involved in a multitude of oncogenic events in human malignancies [2, 3], the most common alteration is partial inactivation of the gene [4–7], suggesting that the normal function of PAX5 is dose dependent. It does, however, appear as if the dose *per se* does not dramatically impact the formation of CD19^+^ cells in mice [8, 9] or induce leukemia, unless combined with additional oncogenic events such as expression of a constitutively active STAT5 [11] or heterozygote deletion of *Ebf* [10] in mouse models. The combined effects of STAT5 activation and PAX5 deficiency is well in line with the observations that activating mutations in in the Il7 or TSLP signaling pathways are frequently observed in human B-ALL [35, 36]. The data presented in this paper suggests additional mechanisms by which multiple oncogenic events may synergize in order to drive malignant transformation in PAX5 mutated B-ALL. The identification of PAX5 PXIs, all representing putative collaboration partners for the protein, suggests that they are frequently mutated in human B-ALLs carrying *PAX5* mutations (Fig 2D). Hence, the transcriptional network around PAX5 can be targeted by multiple mutations that may serve to aggravate the impact on gene expression. It is also notable that mutations in the identified PXIs appear to be enriched in lymphoid malignancies (S3 Fig). Even though we did not observe direct malignant transformation by the combination of ectopic expression of ETV6-RUNX1 or oncogenic forms of IKZF1 and heterozygote deletion of *Pax5* in normal mouse Pre-B cells, this had a profound impact on target gene expression. Based on this we do believe that multiple targeting of proteins being involved in the same regulatory network may be an important part of the oncogenic process.

One general idea of the malignant conversion process is that initial mutations arise in early progenitor cells and that additional mutations accumulate during the differentiation process resulting in the generation of fully developed leukemia in lineage restricted progenitors [37]. This model is well in line with our data however, the ability of PAX5 to direct factors such as RUNX1 and IKZF1 to sites epigenetically silent in the *Pax5*^*-/-*^ mouse Pre-B cells (Fig 3C) opens for other mechanisms possibly contributing to the ability of a modified protein to cause transformation in a lineage restricted manner. Both IKZF1 and RUNX1 are expressed in and impact blood cell development outside of the B-lymphoid compartment [38–42] (S5 Fig) and genetic alterations in early progenitors could therefore result in expression of the oncogenic protein in early multipotent progenitors as well as other lineages. However, since the target gene spectra of the oncogenic proteins could be modified by a B-cell restricted factor such as PAX5 their impact on cellular function may be lineage specific. Hence, the molecular context of the B-cell progenitors could possibly activate the oncogenic potential of mutated proteins by targeting them to epigenetically silenced target sites.

In all, existing data creates a strong link between the level of functional PAX5 activity and malignant transformation. We believe that increased understanding of the regulatory network coordinated by PAX5 will aid in our understanding of how apparently modest disruptions in the regulatory circuitry can contribute to catastrophic events such as malignant transformation and in the long term open novel avenues for diagnosis and targeted treatments.

## Materials and methods

### Please see S1 Text for details

#### Ethics statement

All work is done in line with the regulations defined by the Swedish national agency responsible for animal welfare "Jordbruksverket". The ethical permit was granted by the Animal Ethics Committee at Linköpings Tingsrätt. Approval number 28–14.

#### Animal models

*Wt*,^,^ *Pax5*^*+/-*^ and *Pax5*^*-/-*^ [8] mice were on C57BL/6 (CD45.2) background.

#### Cells and cell culture

Primary fetal liver (FL) Pre-B cells were cultured *in vitro* on OP9 stroma cells using Opti-MEM (ThermoFisher Scientific, Waltham, MA) supplemented with 10% heat-inactivated fetal calf serum (FCS) as in [43]. The mouse Pre-B cell line 230–238 and human B-ALL NALM6 cells were cultured in RPMI1640 with UltraGlutamine (Lonza, Basel, Swiss) supplemented with 10% heat-inactivated FCS, 20mM HEPES, 50μg/ml Gentamicin and 50μM β-ME.

#### BioID assay

Human PAX5 as well as an SV40 NLS encoding cDNA was ordered from GenScript (Piscataway, NJ) and sub-cloned into retroviral pMIG vector carrying BIRA*. Retroviruses were produced and used to infect 230–238 mouse Pre-B cells. After infection, sorted GFP^+^ cells were pulsed with 50μM d-biotin. Following biotinylation, cells were frozen in -80°C before being resuspended in lysis buffer for sonication. Pull down was achieved when the cell lysate was incubated with streptavidin-sepharose beads followed by trypsination. Tryptic peptides were analyzed on a reverse phase nano liquid chromatography coupled online to an LTQ Orbitrap Velos Pro (ThermoFisher Scientific) mass spectrometer. Identification and quantitation were achieved using Proteome discoverer (Thermo Scientific, version 1.3), SEQUEST algorithm (Thermo Fisher Scientific, San Jose, CA, USA; version 1.4.0.288) and X! Tandem (CYCLONE (2010.12.01.1) Data analysis was reconfirmed using Trans-Proteomic Pipeline (TPP) software [44] and Prohits software suite generated valid interactions with a SAINT score A Bayesian FDR of 0.02 (corresponding to a SAINT score of ~0.80) was used as a cut-off to define high confidence interactors.

PXIs were analyzed by PANTHER Overrepresentation Test (release 2018/10/10) (http://geneontology.org/), and enrichment analyses were run with Gene ontology database released on 2018/9/6. Differentially Up or Downregulated genes were subject to GO-term analysis using The Database for Annotation, Visualization and Integrated Discovery (DAVID) v6.8 (https://david.ncifcrf.gov/) [45, 46].

#### RNA-sequencing and data analysis from cultured transduced Pre-B cells

RNA was prepared from primary mouse FL Pre-B cells from *Wt*, and *Pax*^*+/-*^ mice transduced with retroviruses encoding functionally impaired IKZF1-protein (IKZF1DN) or ETV6-RUNX1 fusion protein. RNA-seq was performed as in [43]. Data was mapped to reference genome mm10 and analyzed using the HOMER package. For details see extended MM.

#### Chromatin immunoprecipitation and ATAC-seq analysis

ChIP-seq and ATAC-seq analysis was performed essentially as in [43]. Briefly, ChIP was carried out using 10 μg per 10^7^ cells of rabbit anti-Ikzf1 [ab26083, Abcam], anti-FLI1 polyclonal IgG [ab15289, Abcam], anti-Ebf1 [ABE1294, Millipore], anti-Pax5 [ab183575, Abcam], anti-RUNX1 polyclonal IgG [ab23980, Abcam] or 10μl of rabbit anti-H3K4Me3 polyclonal IgG [07–473 Millipore], or 25μg of rabbit anti-H3K27Ac IgG [ab4729, Abcam]. NALM6 ChIP-seq data peaks were called with and HOMER adapted version of the IDR package (Irreproducibility Discovery Rate) package [47] (Karmel A. 2015. homer-idr: Second pass updated) according to (https://sites.google.com/site/anshulkundaje/projects/idr).

For details on ChIP-seq or ChIP-QPCR see extended MM.

#### Proximity Ligation-Assisted ChIP (PLAC)-sequencing

PLAC-seq was carried out and analyzed in duplicates similar to previously reported [15] with minor modifications. Approximately, 250M valid H3K4me3 interactions pairs and 310M valid H3K27ac interaction pairs were generated. Bias corrected significant interactions (FDR ≤ 0.05) between anchor points and other anchor points/non-anchor-points (peak-to-all) were identified with the FitHiChIP pipeline (https://www.biorxiv.org/content/early/2018/09/10/412833). Interactions shared between samples within 1 bin size (5kb) were merged with a custom adaption of the *merge2Dbed*.*pl* from the HOMER platform [48]. Interaction anchor points were annotated against *hg19* with a custom bash script utilizing the HOMER annotation database (*annotatePeaks*.*pl*). A custom bash script utilizing *Bedtools intersect* [49] was used to derive interactions overlapping transcription factor ChIP-seq peaks in either or both end-points. For details see extended MM.

#### RNA-seq analysis of primary human B-ALL

We extracted normalized RNA-seq data from two previously described patient cohorts [28] and part of the B-ALL phase II dataset [6] accessible in the TARGET repository. PAX5, RUNX1 and IKZF1 ChIP-seq peaks from NALM6 cells were annotated against hg19 with *annotatePeaks*.*pl* from the HOMER platform [48]. Peaks within 2.5kb (upstream or downstream) of TSS were assigned to the closest genes with proximity-based annotations. To assign a distal TF peaks (more than 2.5 kb away from a TSS) to genes the NALM6 H3K4me3 PLAC-seq interactions were utilized using a custom R script. TF sites further from TSS than 2.5kb that did not overlap with an interaction were discarded. Genes were considered co-bound by two transcription factors if they were annotated to the same gene independent of their position in the gene. Bound genes for each category (PAX5, RUNX1, IKZF1, PAX5-RUNX1, PAX5-IKZF1) were tested for differential expression with DESeq2 [50] between mutated (PAX5, RUNX1 or IKZF1) and unmutated B-ALL cases (Fig 5A–5C) or double-mutated (PAX5-RUNX1 or PAX5-IKZF1) and PAX5 single-mutated B-ALL cases (Fig 5D and 5E). Only genes with 10 or more counts in at least two samples were included in the analysis. Samples with missing mutation information were excluded for a given comparisons. Mean of log2 normalized RPKM for differentially expressed genes between two conditions were plotted as boxplots and significance between categories were tested with Mann-Whitney *U*-test.

#### Data availability

In line with the data availability policy, ChIP-, RNA-, ATAC- and PLAC-sequencing data generated for this paper are freely available and have been deposited in GEO under the acc. Numbers; GSE126375 for murine data and GSE126300 for data on the human cell-line NALM6.

## Supporting information

S1 Text Extended supplementary materials and methods. This supplement contains detailed protocols for the methods used in this report.
(DOCX)

S1 Table Identification of PAX5 proximity interactors (PXIs) in Pre-B cells. The diagram displays proteins identified as PAX5 PXIs in 230–238 mouse B-ALL cells. The Saint score and the enrichment of a given protein as compared to what was observed in cells transduced with an NLS-BIRA* is indicated. Proteins previously identified as PAX5 interacting factors [16] or proteins identified by STRING analysis (https://string-db.org) as direct or indirect partners (S1 Fig) are indicated. The table also display GO-terms defined using Gene ontology (GO) analysis performed with PANTHER14.0.
(XLSX)

S2 Table PAX5, RUNX1 and IKZF1 display overlapping binding at putative regulatory elements annotated to defined genes. The table display genes annotated to specific ChIP-seq peaks with unique or combined binding of transcription factors as shown in Fig 3A. Genes annotated to several categories were excluded from the analysis.
(XLSX)

S3 Table PAX5, RUNX1 and IKZF1 target genes involved in the regulation of cell proliferation and transcription. The table display a GO analysis (DAVID v6.8 (https://david.ncifcrf.gov/)) [45, 46] of genes annotated to specific ChIP-seq peaks with unique or combined binding of transcription factors (S2 Table) as shown in Fig 3A. Genes annotated to several categories were excluded from the analysis.
(XLSX)

S4 Table PAX5, RUNX1 and IKZF1 display overlapping binding at putative regulatory elements annotated to defined genes. The table display genes annotated to specific ChIP-seq peaks with unique or combined binding of transcription factors as shown in S4D Fig. Genes annotated to several categories were excluded from the analysis.
(XLSX)

S5 Table PAX5, FLI1 and IKZF1 target genes involved in the regulation of cell proliferation and transcription. The table display a GO analysis (DAVID v6.8 (https://david.ncifcrf.gov/)) [45, 46] of genes annotated to specific ChIP-seq peaks with unique or combined binding of transcription factors (Table 4) as shown in S3D Fig. Genes annotated to several categories were excluded from the analysis.
(XLSX)

S6 Table Differential gene expression patterns upon expression of ETV6-RUNX1 or DN-IKZF1 in Wt or *Pax5^+/-^* mouse Pre-B cells. Differentially expressed genes between *Wt* and *Pax5*^*+/-*^ Pro-B cells transduced with either constructs encoding TEL-AML or IKZF1-DN compared to control vector (pMIG) transduced counterparts as indicated were identified by RNA-seq as described in materials and methods and extended methods. Log2 fold change, p-Value and adjusted p-Value (FDR) from the output file of the *getDiffExpression*.*pl* (using edgeR as statistical tool) command, run in HOMER are listed for each comparison in the form of a table listing all 24072 examined genes.
(XLSX)

S7 Table Differential gene expression patterns upon expression of ETV6-RUNX1 or DN-IKZF1 in normal or *Pax5^+/-^* cells. Differentially Up or Downregulated genes defined in S6 Table were subject to GO-term analysis using The Database for Annotation, Visualization and Integrated Discovery (DAVID) v6.8 (https://david.ncifcrf.gov/) [45, 46].
(XLSX)

S8 Table A fraction of the PAX5, IKZF1 and/or RUNX1 target genes are differentially expressed in tumors carrying transcription factor mutations. The tables show the identity as well as the expression levels of transcription factor target genes in normal and malignant cells identified as up or down regulated by DESeq2 [50] in correlation to mutation in the targeting transcription factor. Genes were identified as in Fig 5A–5E.
(XLSX)

S9 Table Identification of biological functions of significantly differentially expressed target genes in primary human leukemia. Differentially Up- or Down-regulated transcription factor target genes identified in Fig 5 were subject to GO-term and KEGG pathway analysis using The Database for Annotation, Visualization and Integrated Discovery (DAVID v6.7 (https://david.ncifcrf.gov/) [45, 46].
(XLSX)

S1 Fig STRING analysis identifies multiple PXIs as potential partners for PAX5. The figure displays a network map generated by analysis of the PAX5 interactome using Search Tool for the Retrieval of Interacting Genes/Proteins (STRING) (https://string-db.org). Colored nodes indicate the query protein (PAX5, Red) and first shell of interactors. Meanwhile, the light grey nodes indicate the second shell of interactors. Edges represent protein-protein associations. The minimum required interaction score was 0.9.
(PDF)

S2 Fig PAX5 is associated with a large variety of transcriptional regulators in pre-B cells. The diagram displays a functional enrichment analysis identified overrepresented protein classes in the dataset by Gene ontology (GO) analysis performed with PANTHER14.0.
(PDF)

S3 Fig Mutations in PAX5 and PXIs is enriched in lymphoid leukemias. The diagram displays the fraction of tumors of different histological origin with reported PAX5 PXI mutations vs total number of reported tumors per type are listed. The analysis was based on a list of 239 PAX5 PXI´s that were investigated in the public cancer database COSMIC (cancer genes sensus V76) obtained from the Sanger Institute Catalogue Of Somatic Mutations In Cancer web site, (http://cancer.sanger.ac.uk/cosmic) [14]. Only entries classified as hematopoietic and lymphoid tissue are considered.
(PDF)

S4 Fig PAX5, IKZF1 and RUNX1 share binding to multiple sites in the mouse Pre-B cell genome. (**A**) Diagrams displaying Q-PCR data for co-precipitations of the *Igll1* and *Cd79a*/*Mb-1* promoters after ChIP of EBF1 (*n* = 7), PAX5 (*n* = 3), FLI1 (*n* = 4), IKZF1 (*n* = 4), and CBF0β (*n* = 3) from 230–238 Pre-B cells. The diagrams are based on the relative enrichment as compared to a polyclonal IgG. Statistical analysis was based on Student’s *t*-tests (two-tailed). ***: *p* < 0.0005 (**B**) Peak files from ChIP-seq data of PAX5, IKZF1 and RUNX1 in 230–238 cells (Fig 3A) were analyzed for motif enrichment using findMotifsGenome.pl in Homer (mm10 -size 200). Rank, enriched motif, P-value, % of target (T) and background (Bg) and best match to known motifs of Top 3 motifs plus PAX5, EBF1 and RUNX1 motif when present for each peak set are listed. (**C**) Diagrams displaying Q-PCR data for co-precipitations of *Igll1* promoter after ChIP-experiments in *Wt*, or *Pax5*^-/-^ pro-B cells. EBF1 (*n* = 5), PAX5 (*n* = 3), FLI1 (*n* = 3), IKZF1 (*n* = 5), and CBFβ (*n* = 3). P-Values were based on Student’s *t*-test (two-tailed) ***: *p* < 0.0005, **: *p* < 0.005, *: *p* < 0.05 RQ: relative quantity. Panel **(D)** display a Venn-diagram of ChIP-seq peaks based on PAX5, IKZF1 and FLI1 ChIP-seq analysis using the 230–238 Pre-B cell line. Peaks were called using the HOMER platform (findPeaks -style factor) and resulting files were filtered for peaks ≥15 normalized tags. Overlapping peaks were identified using the mergePeaks command in HOMER.
(PDF)

S5 Fig Combined binding of PAX5, IKZF1 and RUNX1 is associated with high levels of gene expression in hematopoietic cells. The schematic diagrams in panel (**A-D**) display Gene Expression Commons generated representation of expression patterns for transcription factors or genes annotated to transcription factor binding in S2 Table (**A-C**) and S4 (**D**). Gene lists were uploaded as csv files and analysis was performed using the Gene-set activity function. Expression levels are indicated with heatmaps reaching from dark Blue (Low-expression) to dark Brown (High-expression) as indicated.
(PDF)

S6 Fig PAX5, EBF1, IKZF1 and RUNX1 share binding to multiple sites in the human pre-B cell genome. Peak files from ChIP-seq data of PAX5, EBF1, IKZF1 and RUNX1 in NALM6 cells (Fig 4A) were analyzed for motif enrichment using *findMotifsGenome*.*pl* in Homer (hg19 -size 200). Rank, enriched motif, P-value, % of target (T) and background (Bg) and best match to known motifs of Top 3 motifs plus PAX5, EBF1 and RUNX1 motif when present for each peak set are listed.
(PDF)

S7 Fig PAX5, EBF1, IKZF1 and RUNX1 are part of an intricate network of regulatory loops. The figure panels (**A-C**) displays WashU Genome Browser tracks of the *EBF1*, *IKZF1* and *RUNX1* genes in NALM6 B-leukemic cells. ChIP-seq data of EBF1, PAX5, RUNX1 and IKZF1 were linked to H3K4me3, H3K27ac chromatin marks and ATAC-seq as well as H3K4me3 and H3K27ac anchored PLAC-seq interactions on the *EBF1* (**A**) *IKZF1* (**B**) and *RUNX1* (**C**) genes. The data is visualized in the WashU Genome Browser.
(PDF)

S8 Fig PAX5, EBF1, RUNX1 and IKZF1 distal interactions are common in human B-leukemic cells. Panel (**A, B**) show diagrams displaying the abundance of combined PAX5, EBF1, RUNX1 and/or IKZF1 binding at identified PLAC-seq anchor-points. Top 40 combinations out of 251 possible for H3K27ac (**A**) and 252 for anchor-point bound H3K4me3 (**B**) PLAC-seq interactions are visualized as UpSet plots. Interactions were filtered for PAX5, EBF1, RUNX1 and/or IKZF1 overlapping ChIP-seq peaks in either one or both anchor-points (for details, see Methods). UpSet plots describe how the combination of TFs in one anchor-point (ap) relates to combination of TFs in the other. Panel (**C-D**) show Chord diagram displaying distal-to-TSS (transcription start site) anchored PAX5-EBF1-RUNX1-IKZF1 gene networks in NALM6 cells. C) H3K4me3 or D) H3K27ac anchored PLAC-seq was used to define chromatin interactions in NALM6 cells. Interactions were filtered for PAX5, EBF1, RUNX1 and/or IKZF1 overlapping ChIP-seq peaks in either one or both anchor-points Anchor points were defined as either distal (more than 2.5kb away from TSS) or as TSS-anchored (within 2.5kb from TSS). The chord diagrams were visualized with the *circlize* package in R. (**E**) ECDF plot displaying the association of PAX5 binding in NALM6 cells and gene expression changes upon introduction of PAX5 in PAX5 mutated REH cells (GSE57480). The following gene categories are shown: Red line describes genes identified as down regulated in patient samples carrying a mutated *PAX5* gene and targeted for PAX5 binding in NALM6 cells (Fig 5A, S8 Table). Blue line shows all genes with PAX5 binding in NALM6 independent of their gene expression status in primary human B-ALL samples (genes in the differentially expressed (red) category were excluded). Black line describes the expression changes of PAX5 unbound genes upon PAX5 reintroduction. Kolmogorov-Smirnov *p*-values are shown.
(PDF)
