# Supplementary material for: PAX5 is part of a functional transcription factor network targeted in lymphoid leukemia
Source: PLoS Genet. 2019 Aug 5;15(8):e1008280. doi: 10.1371/journal.pgen.1008280 (PMC6695195; doi:10.1371/journal.pgen.1008280)
Supplement: S1 Text — This supplement contains detailed protocols for the methods used in this report. (DOCX) [file pgen.1008280.s001.docx]

**Extended supplementary materials and methods**

**PAX5 is part of a functional transcription factor network targeted in lymphoid leukemia.**

Kazuki Okuyama^1^, Tobias Strid^1,2^, Jacob Kuruvilla^1,2^, Rajesh Somasundaram^1^ Susana Cristobal^1^, Emma Smith^2^, Mahadesh Prasad^1^, Thoas Fioretos^3^, Henrik Lilljebjörn^3^, Shamit Soneji^2^, Stefan Lang^2^, Jonas Ungerbäck^&2^ and Mikael Sigvardsson^&1,2*^.

**Extended supplementary materials and methods**

***Animal models.***

*Wt,^,^ Pax5^+/-^* and *Pax5^-/-^* (1) mice were on C57BL/6 (CD45.2) background. Animal procedures were performed with consent from the local ethics committee at Linköping University (Linköping, Sweden).

***Cells and Cell culture.***

Fetal liver (FL) pro-B cells were cultured *in vitro* on OP9 stroma cells using Opti-MEM (ThermoFisher Scientific, Waltham, MA) supplemented with 10% heat-inactivated fetal calf serum (FCS) (HyClone, GE Healthcare, Chicago, IL), 25mM HEPES (ThermoFisher Scientific), 50μg/ml Gentamicin (ThermoFisher Scientific), 50μM β-mercaptoethanol (β-ME) (Sigma-Aldrich, St. Louis, MO), 10ng/ml KIT ligand, 10ng/ml FLT3 ligand, and 10ng/ml Interleukin-(IL-7). All mouse cytokines were bought from PeproTech (Rocky Hill, NJ). The murine Abelson virus-transformed cell line 230-238, were cultured in RPMI1640 with UltraGlutamine (Lonza, Basel, Swiss) supplemented with 10% heat-inactivated FCS, 20mM HEPES, 50μg/ml Gentamicin and 50μM β-ME. NALM6 were cultured in RPMI-1640 with UltraGlutamine (Lonza, Basel, Swiss) supplemented with 10% heat-inactivated FCS, 20mM HEPES, 50μg/ml Gentamicin and 50μM β-ME.

***BioID assay.***

PAX5 encoding cDNA was ordered from GenScript (Piscataway, NJ) and sub-cloned into retroviral MigR1 vector carrying BIRA* which resulted in the fusion of BIRA* to the C-terminal of bait proteins. As a control protein, we employed BIRA* harboring NLS from SV-40 on its N-terminus. Constructed vectors were transfected into Phoenix cells for viral production by use of XtremeGene HP DNA transfection reagent (Roche, Basel, Swiss) according to manufacturer’s protocol. Conditioned medium was harvested 2 days after the transfection. Subsequent retroviral infection of 230-238 cells was performed by spin infection (1,800 x *g*, for 90 min, at 32 °C). Three days after the infection, GFP^+^ cells were sorted with BD FACSAriaIII (Becton, Dickinson and Company, Franklin Lakes, NJ), and propagated in *vitro*. According to (2), when the cells multiply to about 70% confluency in 150 mm culture flasks, d-biotin (Sigma-Aldrich, B4501-1G) was added to a final concentration of 50µM. Cells were incubated for a maximum of 24 hours at 37 ºC. About 100 million cells were pooled, washed twice in ice-cold PBS and frozen in liquid nitrogen. They were stored in -80 ºC till further analysis.

The cells were resuspended in 10 ml lysis buffer (25mM Tris-HCl [pH 7.6], 150mM NaCl, 1% sodium deoxycholate, 0.1% SDS, protease and phosphatase inhibitor cocktail tablets [ThermoFisher Scientific, 88669]) and benzonase nuclease (Sigma-Aldrich, E1014-5KU)) and sonicated (@ 23 kHz, 105 μm, for 3 x 10s on a Soniprep 150 (MSE UK)) to breakup cell clumps and finally incubated for an hour on an end-over-end rotator at 4 ºC. The samples were then transferred into a centrifugation tube and spun at 4 ºC and 16,000 rpm for 30 minutes to get rid of cell debris. Meanwhile streptavidin-sepharose beads (GE healthcare, 17-5113-01) (30 µl of packed bead volume) were washed and equilibrated in the lysis buffer by spinning them at 2000 rpm for 2 minutes. These beads were then suspended into supernatants from the cell lysates and incubated at 4 ºC on a laboratory rocker for 3 hours so as to induce effective conjugation of biotinylated residues and streptavidin followed by 3 rounds of washing in 50mM Ammonium bicarbonate to minimize non-specific binding. The beads were transferred into another tube and washed again 2 times. The biotin conjugated beads were then re-suspended in 50mM ammonium bicarbonate with 2 µl of 1µg/µl trypsin (~20U, ThermoFisher Scientific, Ref# V5113). The tubes were then sealed and incubated overnight at 37 ºC on an end-over-end rotator. 1 µl of 1µg/µl trypsin was added again the next day to achieve effective trypsination. The supernatant was saved in a fresh tube and the beads were washed with fresh 50 mM ammonium bicarbonate and later pooled. The digests were then dried in a vaccum dryer (Savant SPD 1010).

***Mass spectrometric analysis.***

Biological triplicates were considered for each sample of which technical duplicates were tested. The dried samples were reconstituted in 0.1 % formic acid and peptide concentration was measured using a NanoDrop (ND 2000, ThermoFisher Scientific). The mass spectrometric analysis was performed on a reverse phase nano liquid chromatography coupled online to a LTQ Orbitrap Velos Pro (ThermoFisher Scientific). Each of the samples were separated using an Agilent 1200 Easy nLC (Agilent Technologies) system with a nano-electrospray ion source (Proxeon). The peptides were trapped using a pre-column (NS-MP-10-C18-Biosphere, 5 µm particle size, 120 Å, 100 µm x 20 cm) and separated on an analytical column column (NS-AC-10-C18-Biosphere, 5 µm particle size, 120 Å, 75 µm x 10.2cm). A linear gradient from 2% to 35% buffer B (0.1 % formic acid in acetonitrile) against buffer A (0.1% formic acid in water) was carried out with a constant flow rate of 300 nL/min and eluted using a 120 minute gradient.

Full scan MS spectra was acquired in the positive mode electrospray ionization with the ion spray voltage pf 2.4 KV, RF lens voltage of 69 and a capillary temperature of 235 ºC. This was acquired over an m/z of 390-2000 Da at a resolution of 60000 and top 20 intense ions were selected for MS/MS under an isolation width of 1 m/z units with a minimum ion count of 100 for activation. Collision energy of 35 was used to fragment the ions in the collision induced dissociation (CID) mode. The selected masses were included in a dynamic exclusion list for 30 seconds with a repeat duration of 30 seconds.

Peptides were analyzed using cycles consisting of a full precursor ion scan followed by 20 product ion scans where peptides are isolated based on their intensity in the full survey scan (threshold of 500 counts) for tandem mass spectrum (MS2) generation that permits peptide sequencing and identification.

***BioID data analysis.***

Proteome discoverer (Thermo Scientific, version 1.3) was used for protein identification and quantitation with the SEQUEST algorithm (Thermo Fisher Scientific, San Jose, CA, USA; version 1.4.0.288) and X! Tandem (CYCLONE (2010.12.01.1). Trypsin was chosen as the enzyme allowing up to 2 missed cleavages; phosphorylation of serine, threonine and tyrosine resides, oxidation of methionine residue, deamidation of Asn and Gln, pyroGlu of the N terminus and acetylation of protein N-terminus were selected. The searches were performed with the precursor ion mass tolerance up to 10 ppm and a fragment ion mass tolerance of 0.6 Da. The database search was performed against the complete mouse database from Uniprot (85828 entries). All searches were done against a decoy database with a false discovery rate (FDR) of less than 0.01. The minimum peptide length considered was 6 and the false discovery rate (FDR) was set to 0.01 for both proteins and peptides.

Data analysis was achieved using Trans-Proteomic Pipeline (TPP) software (3) from Prohits software suite (4). Vendor specific, Thermo ‘.raw’ files were converted into open format, ‘.mzXML’ files using proteowizard (5) and database searches were performed by comet (6). Proteins identified with a ProteinProphet cut-off of 0.85 (corresponding to ≤1% FDR) and with ≥ 2 unique peptides were analyzed with SAINT Express v.3.3. Each biological replicate was analyzed considering two technical replicates. Data were compared to 6 controls (3 biological and 2 technical replicates of each) with BirAM conjugated to NLS collapsed to the 2 highest spectral counts for each prey. A Bayesian FDR of 0.02 (corresponding to a SAINT score of ~0.80) was used as a cut-off to define high confidence interactors. Prohits lite (version 3.0.3) were run on Linux Fedora virtual machine in Oracle VM ware (version 5.0.2 r 102096). Output from TPP for each sample served as input to the prohits analyst stand-alone application. The sample files were uploaded according to their respective baits and experiments were analyzed. The search results were further analyzed using a statistical tool, SAINT with 5000 iterations, 1 min fold and low mode off along with normalization to calculate interaction confidence scores which was also integrated into Prohits.

***Gene ontology and proteomics analysis.***

PXIs of each transcription factor were uploaded to PANTHER Overrepresentation Test (release 2018/10/10) (<http://geneontology.org/>), and enrichment analyses were run with Gene ontology database released on 2018/9/6. *Mus musculus* genes in the database (22320 genes) were employed as reference. PXIs with transcription factor, coactivator, corepressor, or histone modification activities, or components of chromatin remodeling complexes were identified according their corresponding GO terms (see figure legends). PAX5- and EBF1-PXIs interactions/associations were visualized with Cytoscape 3.3.0 (7).

***RNA-sequencing and data analysis from cultured transduced pro-B cells.***

FL cells from *Wt*, and *Pax^+/-^* FL cells from *Wt*, and *Pax^+/-^* transduced with functionally impaired IKZF1-protein (IKZF1DN) or ETV6-RUNX1 fusion protein were expanded on the stroma cell line OP9 (8). IKZF1DN, lacking all the four Zn-fingers in the DNA binding domain due to deletion of coding exon 3-5 were synthesized by Genscript. ETV6-AML1 was generated by fusing human ETV6 exon 1-5 (correspond to 275-1283 nucleotides from NM_001987.4) and human RUNX1 exon 2-8 (correspond to 249-1630 nucleotides from NM_001754.4). This construct was subcloned into EcoRI site on MigR1 vector. Total RNA was isolated using RNAeasy Micro Kit (Qiagen, Hilden, Germany) and libraries constructed using NuGEN’s Ovation Ultralow Library systems (NuGEN Technologies, San Carlos, CA). RNA-seq libraries were subject to 76 cycles single end sequencing on a NextSeq500 (Illumina, San Diego, CA). Reads were aligned to mouse reference genome (mm10 / GRCm38) using STAR (2.6.0b-1) (9) and downstream analyses were performed using the HOMER platform (v4.8 and v4.10)(10). For analysis of statistically significance among differently expressed genes the data was analyzed using *analyzeRepeats.pl* with the *–noadj* option followed by the *getDiffExpression.pl* command using edgeR (3.12.0)(11).

***Chromatin immunoprecipitation.***

Cells for transcriptions factor ChIP were fixed at RT in 1 mg/ml DSG (ThermoFisher Scientific) in PBS for 30 min followed by an additional 10 min after addition of formaldehyde up to 1%. Cells for Histone modification ChIP were fixed in 1% formaldehyde for 10 min. The reactions were quenched by addition of 1/10 volume of 0.125M glycine and the cells were washed in PBS. Nuclei were isolated by 10 min incubation in Nuclei Isolation buffer (50 mM Tris-pH 8.0, 60 mM KCl, 0.5% NP40) + protease inhibitor cocktail (PIC) (1X Roche protease inhibitors – 11697498001) on ice. Pelleted nuclei were dissolved in Lysis buffer (0.5% SDS, 10 mM EDTA, 0.5 mM EGTA, 50 mM Tris-HCl (pH 8)) + PIC and sonicated on a Bioruptor (Diagenode). Sonication was followed by pelleting of debris and the supernatant was transferred to new tube and chromatin was diluted 5X in Dilution Buffer (1% Triton, 2mM EDTA, 150 mM NaCl, 20 mM Tris-HCl (pH 8) + PIC) for transcription factor ChIP and in HBSS (Lonza, Verviersa, Belgium) +PIC and 2X RIPA buffer (20 mM Tris–HCl, pH 7.5, 2 mM EDTA, 2% Triton X-100, 0.1% SDS, 0.2% Sodiumdeoxycholate, 200 mM NaCl) + PIC for histone modification ChIPs. Ten μg per 10^7^ cells of antibody Rabbit anti-IKZF1 polyclonal IgG [ab26083, Abcam], anti-FLI1 polyclonal IgG [ab15289, Abcam], Rabbit polyclonal anti-Ebf1 [ABE1294, Millipore], Rabbit polyclonal anti-PAX5 [ab183575, Abcam], Rabbit polyclonal anti-RUNX1 [ab23980, Abcam] or 10μl of H3K4Me3 polyclonal IgG [07-473 Millipore], or 10μg of Rabbit polyclonal anti-H3K27Ac IgG [ab4729, Abcam] was hybridized to 70μl Protein-G or A Dynabeads (Life Technologies). ChIP was performed over night at 4°C, and subsequently washed (1 time with 500 μl Low Salt Immune Complex Wash Buffer, 1 time with 200 μl High Salt Immune Complex Wash Buffer, 1 time with 200 μl LiCl Immune Complex Wash Buffer, 2 times with 200 μl TE buffer) and eluted for 6 h at 65°C (20 mM Tris-HCl, pH 7.5, 5 mM EDTA 50 mM NaCl, 1% SDS, 100 μg RNase A and 50 μg proteinase K) treated and finally cleaned up using Zymo ChIP DNA Clean & Concentrator before ChIP-qPCR or ChIP-seq library preparation using NEXTflex DNA barcodes (BIOO scientific). 76 bp single read sequencing was performed on an Illumina NextSeq500. NALM6 transcription factor and histone ChIPs were carried out and analyzed in duplicates.

***ChIP-qPCR.***

DNA fragments of *Alb* (NC_000071.6 [90450047..90450144]), *Igll1* (NC_000082.6 [16864173..16864274]) and *Cd79a*/Mb1 (NC_000073.6 [24897304..24897409]) were quantified by real-time qPCR with FastStart Universal SYBR Green Master (Rox) (Roche) after the ChIP. Designed primers are as follows; *Alb* forward: acctgcgttacagcatccac, *Alb* reverse: tgctgacagagcaggagaca, *Igll1* forward: ggcaggtgttcagttgctct, *Igll1* reverse: gcagctctgcctgactgata, *Cd79a* forward: cacgcactagagagagactcaag, and *Cd79a* reverse: ccttactcctggccctttatttgcc. Threshold cycle (Ct) of target fragment was normalized with Ct of *Alb* fragment, and ΔΔCt algorithm against control IgG was employed for the relative quantification. Rabbit Rabbit anti-FLI1 polyclonal IgG [ab15289, Abcam, Cambridge, UK], Rabbit anti-IKZF1 polyclonal IgG [ab26083, Abcam], Rabbit anti-CBFβ polyclonal IgG [ab125191, Abcam], or Rabbit anti-IgG polyclonal IgG [ab46540, Abcam] [as control IgG])

***ChIP-seq data-analysis***

Reads from high throughput sequencing of murine cells were aligned to mouse reference genome mm10 using Bowtie2 (2.3.4.3) (12). Further analyzes were performed using the HOMER package (v4.8 and v4.10)(10). For FL and 230-238 ChIP-seq analysis, transcription factor peaks were identified using *findPeaks.pl* with the *–style factor* parameter and normalized to seq- inputs. For the human NALM6 cell line ChIP-seq analysis, reads were aligned to the human reference genome hg19 using Bowtie2, transcription factor peaks were identified with *findPeaks.pl* against a matched control sample using the settings “*-P .1 -LP .1 -poisson .1 -style factor*”. Transcription factor peak reproducibility was determined by a HOMER adaptation of the IDR (Irreproducibility Discovery Rate) package (13) (Karmel A. 2015. homer-idr: Second pass updated) according to (https://sites.google.com/site/anshulkundaje/projects/idr). Only reproducible high-quality peaks, defined by normalized scores of at least 10 tags/10 million and an acceptable IDR score, were submitted to further analysis.

Motif enrichment analysis was performed with the *findMotifsGenome.pl* command of the HOMER package. Overlapping peaks between samples were identified using *mergePeaks.pl* (default parameters). Tag density plots and heat maps were created with *annotatePeaks.pl* (*–hist* or *–hist* & *-ghist* respectively), normalizing data to 10 million mapped reads per experiment, and visualized using Excel or by hierarchical clustering in Cluster3 (v3.0) (14) followed by Java Treeview (v.1.1.6r4) (15). UCSC genome browser was used for visualization of BedGraph files (16).

Annotation of ChIP-seq peaks to specific genes by proximity was perfomed using *annotatePeaks.pl* (mm10) in HOMER. Go-term analysis of genes identified by ChIP-peak annotation was perfomed in DAVID Bioinformatics Resources 6.8 NIAID/NIH (david.ncifcrf.gov) (17) and (18) by uploading gene name lists.

Expression analysis of defined gene sets were performed using Gene Expression Commons Gene set analysis tool. Gene lists defined as above were converted into csv files to be uploaded for the formation of specific gene sets. Expression patterns were generated using the mouse hematopoiesis model and the results were exported as pdf files for compilation into Figure S5.

Previously published sequencing data used in analyses was retrieved from GEO (GSE92434) (19).

***Assay for Transposase Accessible Chromatin (ATAC-seq)***

Processing of samples for ATAC-seq library preparation was performed essentially as described in (20). Libraries were single-end sequenced for 76 cycles on a NexSeq500.

***Analysis of ATAC-seq data***

Mouse data was mapped to mm10 using Bowtie2 (Galaxy Version 2.3.4.2) (12, 21) with standard settings. Tag directories with reads mapped to the mitochondrial chromosome filtered out and UCSC BedGraph files normalized to 10M total mapped reads were created using the HOMER platform (10) (*makeTagDirectory, makeUCSCfile)*. BedGraph files were up-loaded to the UCSC-genome browser (16) for visualization. Abundance of ATAC-tags on transcription factor ChIP-seq peaks was analyzed in HOMER using the *annotatePeaks.pl* command using normalization to 10M mapped reads.

NALM6 ATAC-seq analysis was performed in triplicates and single-end processed with the ENCODE ATAC-Seq/DNase-Seq Pipeline (<https://github.com/kundajelab/atac_dnase_pipelines>) with the following settings: *-species hg19 -no_xcor -enable_idr -auto_detect_adapter -out_dir out_NALM6_standard_se -rm_chr_from_tag mito -no_random_source -multimapping 4 -nth 8 -se*. BigWig files were generated from deduplicated bam-files as follows: Briefly, the tag directory converted to the bed format using *tag2bed.pl* from the HOMER package (10) and sorted with *Bedtools sort* followed by *Bedtools genomeGoverageBed* (normalized to 10 million reads) (22) and UCSC *bedGraphToBigWig* and visualized on the WashU EpiGenome Browser (23).

***Proximity Ligation-Assisted ChIP (PLAC)-sequencing.***

PLAC-seq was carried similar to previously reported with minor modifications (24). 10M NALM6 cells that had been cross-linked with 1% formaldehyde for 10 minutes (see *Chromatin immunoprecipitation* section) were thawed on ice for 5 minutes, re-suspended in ice cold lysis buffer (10 mM Tris-HCl pH 7.5, 10 mM NaCl, 0.2% NP-40, 1X Roche protease inhibitors – 11697498001 (PIC)) and incubated on ice for 15 min. Samples were spun for 5 min @ 4°C at 2,500g and cell pellet was washed in ice-cold lysis buffer + PIC. The pellet was resuspended in 0.5% SDS and incubated @ 62°C for 10 min. The SDS reaction was quenched by addition of water and 10% Triton X-100 followed by 15 min incubation @ 37°C. The samples were digested with 40U MboI restriction enzyme with addition of 25 µl NEBuffer2 followed by 2h incubation @ 37°C with shaking (900 RPM). After restriction enzyme digestion the samples were incubated for 20min @ 62°C to inactivate MboI followed by a cool-down to RT. A fill in reaction was conducted using 0.3 mM Biotin-14-dATP (ThermoFisher, 19524016), 0.3 mM dCTP, 0.3 mM dTTP, 0.3 mM dGTP and 40U Klenow (NEB, M0210) @ 37°C for 1.5h with shaking (900 RPM). Next a Ligation master mix (1X T4 ligation buffer (NEB, B0202), 1% Triton-X 100, 120 ug BSA (NEB, B9000), 4000 U T4 DNA ligase (NEB, M0202)) was added and samples were incubated @ RT with rotation for 2h. The samples were centrifugated 2,500g 5min @ 4°C, supernatant was removed and cell pellet was resuspended in 250 µl ChIP SDS-lysis buffer (0.5% SDS, 10 mM EDTA, 0.5 mM EGTA, 50 mM Tris-HCl (pH 8)) + PIC followed by 18 rounds of sonication (30s max intensity followed by 30s rest) with a Bioruptor (Diagenode). Sheared chromatin was centrifuged for 10 min @ 4°C, 13000 rpm and supernatants were transferred to new tubes and diluted in 750 µl 1xHBSS + 1 ml 2X RIPA (20 mM Tris–HCl, pH 7.5, 2 mM EDTA, 2% Triton X-100, 0.1% SDS, 0.2% sodium deoxycholate, 200 mM NaCl) + PIC. 1% input was removed from supernatant, and 10 µg H3K4me3 (Millipore, 07-473) or 10 µg H3K27ac (Abcam, ab4729) antibody adsorbed to 60 µl ProteinG dynabeads (ab adsorption to beads as in ChIP-seq protocol) were added to the remaining supernatant and incubated @ 4°C overnight with rotation. Samples were washed as follows: 2 times with 1 ml Low Salt Immune Complex Wash Buffer (0.1% SDS, 1 % Triton X-100, 2 mM EDTA, 50 mM Tris-HCl pH8, 150 mM NaCl), 2 times with 1 ml High Salt Immune Complex Wash Buffer (0.1% SDS, 1 % Triton X-100, 2 mM EDTA, 50 mM Tris-HCl pH8, 500 mM NaCl), 1 time with 1 ml LiCl Immune Complex Wash Buffer (0.25 M LiCl, 1% Igepal-CA630, 1% sodium deoxycholate, 1 mM EDTA, 10 mM Tris-HCl pH8), 2 times with 1 ml TE buffer (10 mM Tris–HCl, pH 8.0, 10 mM EDTA) followed by elution of chromatin from magnetic beads with two rounds of 100 µl elution buffer (1%SDS, 100 mM NaHCO_3_) with shaking (1500 RPM) @ RT. Supernatants were transferred to new tubes and chromatin complexes were reverse cross-linked overnight @ 65°C with the addition of 250 mM NaCl, 100 μg RNase A (ThermoFisher, EN0531) and 50 μg proteinase K (ThermoFisher, AM2546) followed by Zymo Research ChIP DNA Clean & Concentrator (BIOSITE-D5205) clean up. 25 μL of Streptavidin T1 beads (Thermo Fisher, 65601) were washed with Tween Wash Buffer (5 mM Tris-HCl pH 8, 0.5 mM EDTA, 1 M NaCl, 0.05% Tween-20) then resuspended in 50 μL of 2X Biotin Binding Buffer (10 mM Tris-HCl pH 7.5, 1 mM EDTA, 2M NaCl). Beads were added to the samples and incubated at room temperature for 15 minutes with shaking. After capture, beads were placed on a magnet and supernatant was discarded. Samples were washed twice by adding 500 μL of Tween Wash Buffer and incubated at 55°C for 2 minutes shaking followed by a 1 time wash in 100 µl 1X T4 DNA ligation buffer. Beads were collected on a magnet and resuspended on 100 µl end-repair mastermix (0.5 mM dNTPs(VWR, E636-40UMOLE), 12U T4 DNA Polymerase (NEB, M0203), 50U T4 Polynucleotide Kinase (NEB, M0201), 5U Klenow (NEB, M0210), 1X NEB T4 DNA ligase buffer) followed by incubation @ RT for 30 min with shaking (900 RPM). 300 µl Tween Wash Buffer was added and beads were placed on a magnet and supernatant was discarded. Samples were washed twice by adding 500 μL of Tween Wash Buffer and incubated at 55°C for 2 minutes shaking followed by a 1 time wash in 100 µl 1X NEB2 buffer. Beads were collected on a magnet and resuspended on 100 µl A-tailing mastermix (0.5 mM dATP (ThermoFisher), 25U Klenow Exo- (NEB, M0212), 1X NEB2 buffer) and incubated @ 37°C for 30 min with shaking (900 RPM). 300 µl Tween Wash Buffer was added and beads were placed on a magnet and supernatant was discarded. Samples were washed twice by adding 500 μL of Tween Wash Buffer and incubated at 55°C for 2 minutes shaking followed by a 1 time wash in 100 µl 1X Fast-Link ligation buffer (Epicenter, LK0750H ) followed by NEXTflex DNA barcode ligation (BIOO scientific) using the Fast-link ligation kit (Epicenter, LK0750H). 300 µl Tween Wash Buffer was added and beads were placed on a magnet and supernatant was discarded. Samples were washed twice by adding 500 μL of Tween Wash Buffer and incubated at 55°C for 2 minutes shaking followed by a 1 time wash in 100 µl 10mM Tris-HCl pH 8.0, resuspended in 45 µl and a 1:1000 dilution was made for qPCR determination of number cycles needed for final PCR amplification. After final PCR, T1 streptavidin beads were collected on a magnet, supernatant transferred to new tubes and Ampure XP beads (x0.8 sample volume) were used to clean up libraries. PLAC-seq libraries were subject to 2X 75 cycles of paired-end sequencing on a NextSeq500.

In parallel NALM6 H3K4me3 or H3K27ac ChIP was carried out using 10M cells as described above (see *Chromatin immunoprecipitation* section), using 10 µg H3K4me3 (Millipore, 07-473) or 10 µg H3K27ac (Abcam, ab4729) antibodies. This data was used to define PLAC-seq anchor points in the interaction analysis.

***PLAC-seq data analysis.***

NALM6 H3K4me3 and H3K27ac experiments were carried out and sequenced in duplicates. To optimize interaction calling fastq-files containing both read 1 and read 2 with their counter parts from the two replicates were combined for a total of 200-250M paired-end reads per sample. Reads were trimmed in pair-end mode with Trim Galore (0.5.0)(--paired --fastqc --clip_R1 10 --clip_R2 10 --three_prime_clip_R1 3 --three_prime_clip_R2 3) (<http://www.bioinformatics.babraham.ac.uk/projects/trim_galore/>). Trimmed paired fastq reads were preprocessed through the HiC-Pro pipeline (v2.9.0) (25), using Bowtie2 (v2.3.4.3) (12, 21) for alignment against hg19 (GRCh37) reference genome, assigning mapped reads to a hg19 MboI restriction map followed by removal of PCR duplicates with Picard tools MarkDuplicates (2.18.23) (https://broadinstitute.github.io/picard/) and subsequently the generation of a list of valid interaction read pairs. Approximately, 250M valid H3K4me3 interactions pairs and 310M valid H3K27ac interaction pairs were generated.

Single-end sequenced fastq files from the NALM6 H3K4me3 or H3K27ac ChIP-seq were used as inputs for the generation of interaction anchor points. Reads were trimmed using Trim Galore in single-end mode (--gzip --fastqc --clip_R1 10 --three_prime_clip_R1 3), and trimmed reads were aligned with Bowtie2 to the hg19 reference genome and tag directories were created from the aligned reads (in bam format (converted from sam using samtools (v1.9) (26))) with the *makeTagDirectory* command from the HOMER platform (10). Prior to peak calling replicate tag directories were merged using *makeTagDirectory* and regions were identified using *findPeaks (-style histone)*. Identified regions were migrated to the bed-format using *pos2bed.pl* and these regions were used as anchor points in the PLAC-seq interaction identification.

Bias corrected significant interactions (FDR ≤ 0.05) between anchor points and other anchor points/non-anchor-points (peak-to-all) were identified with the FitHiChIP pipeline (<https://www.biorxiv.org/content/early/2018/09/10/412833>) using the HiC-Pro valid pairs and identified ChIP-anchors as input files with following settings: GCSize (size of the window upstream and downstream the restriction site used to calculate the GC content)=200, MappSize (size of the window upstream and downstream the restriction site used to calculate the mappability)=500, BINSIZE (Size of the bins in bases, for detecting the interactions.)=5000, LowDistThr (Lower distance threshold of interaction between two segments)=10000, UppDistThr (Upper distance threshold of interaction between two segments)=3000000, BiasCorrection with Coverage specific bias.

Interactions shared between samples within 1 bin size (5kb) were merged with a custom adaption of the *merge2Dbed.pl* from the HOMER platform(10) (https://github.com/stela2502/LoopBed). Interaction anchor points were annotated against *hg19* with a custom bash script utilizing the HOMER annotation database (*annotatePeaks.pl*).

A custom bash script utilizing *Bedtools intersect* (22) was used to derive interactions overlapping transcription factor ChIP-seq peaks in either or both end-points.

TF overlapping interactions were imported in to R 3.5.0 and visualized as Upset plots with the UpSetR package (v1.3.3 (27)) or as cord diagrams with the circlize package (v0.4.5(28))

Interactions were uploaded and visualized on the WashU EpiGenome Browser (23).

For the NALM6 transcription factor and H3K4Me3/H3K27Ac (used for PLAC-seq analysis (see below)) ChIP, BigWigs were generated as described in the ATAC-seq analysis Method section. UCSC genome browser (16) or WashU EpiGenome Browser (23) were used for visualization of BigWig files. Code is available at https://github.com/stela2502/LoopBed and <https://github.com/jonasungerback/sigvardsson_lab_plac_tools>.

***RNA-seq analysis of primary human B-ALL.***

The patient cohort included in the study have been described previously (29) (30). For the in-house generated dataset (including healthy bone-marrow control cells), paired end 101 bp reads were aligned to the human reference genome hg19 using Tophat 2.0.7. Raw reads were counted using featureCounts (overlapping genes as described by Gencode v19) from the subread package (1.5.0) (n = 162) (29) or the B-ALL phase II dataset (30) accessible in the TARGET repository downloaded from the TARGET-database ( <ftp://caftpd.nci.nih.gov/pub/OCG-DCC/TARGET/ALL/mRNA-seq/Phase2/L3/expression/BCCA/>) (n = 102). Gene expression was also calculated as reads per kilobase of transcript per million reads (RPKM) from the raw counts using gene lengths derived from the HOMER gene annotation database (10). After merging the datasets and filtering for expressed genes (10 reads in a minimum of 2 samples), 19546 genes remained and were subjected for further analysis.

NALM6 PAX5, RUNX1 and IKZF1 ChIP-seq peaks were annotated against hg19 with *annotatePeaks.pl* from the HOMER platform (10). Peaks within 2.5kb (upstream or downstream) of TSS were assigned to the closest genes with proximity-based annotations. To assign a distal TF peaks (more than 2.5 kb away from a TSS) to genes the NALM6 H3K4me3 PLAC-seq interactions were utilized. Briefly, using a custom R script, TF overlapping interactions were filtered so all remaining interactions had one anchor point in proximity to a TSS and the other in a distal element containing a TF binding site. The distal TF site was then assigned to the interaction connected TSS. TF sites further from TSS than 2.5kb that did not overlap with an interaction were discarded. Genes were considered co-bound by two transcription factors if they were annotated to the same gene independent of their position in the gene. Bound genes for each category (PAX5, RUNX1, IKZF1, PAX5-RUNX1, PAX5-IKZF1) were tested for differential expression with DESeq2 (v 1.22.0) (31) between mutated (PAX5, RUNX1 or IKZF1) and unmutated B-ALL cases (Fig. 5A-C) or double-mutated (PAX5-RUNX1 or PAX5-IKZF1) and PAX5 single-mutated B-ALL cases (Fig. 5D-E). Only genes with 10 or more counts in at least two samples were included in the analysis. Samples with missing mutation information were dropped for a given comparisons. Additional genetic information was not considered in the analysis. Mean of log2 normalized RPKM for differentially expressed genes between two conditions were plotted as boxplots and significance between categories were tested with Mann-Whitney *U*-test. Numbers are indicated in Figure 5.

***ECDF plot generation.***

Raw RNA-seq read counts from PAX5-deficient human B-ALL REH cells comparing doxycycline-induced PAX5 or GFP CTRL-vector expression were downloaded from GEO (GSE57480) (32). Log_2_ fold changes between PAX5 -and CTRL transduced cells were calculated with edgeR (v 3.26.3) using R 3.6.0. Genes lacking expression in both sample categories were excluded from further analysis. The Empirical cumulative distribution (ECDF) plot (Fig. S8E) was generated with the R package ggplot2 (v 3.1.1). The three gene categories in the ECDF plot were determined as follows: 1) Genes with no detectable PAX5 binding in NALM6 cells (black line in Fig. S8E). 2) Genes bound by PAX5 in NALM6 cells (with category 3 genes excluded) (blue line in Fig. S8E). 3) Genes bound by PAX5 in NALM6 cells and down-regulated in PAX5-mutated B-ALL (Fig. 5A) (red line in Fig. S8E). Statistical analysis was performed with a Kolmogorov-Smirnov (K–S) two-sided test.

**Data availability**

ChIP-, RNA-, ATAC- and PLAC-sequencing data generated for this paper have been deposited in GEO under the acc. Numbers; GSE126375 for murine data and GSE126300 for data on the human cell-line NALM6.

**References:**

1. Urbánek P, Wang Z-Q, Fetka I, Wagner EF, Busslinger M. Complete block of early B cell differentiation and altered patterning of the posterior midbrain in mice lacking Pax5/BSAP. Cell. 1994;79:901-12.

2. Dingar D, Kalkat M, Chan PK, Srikumar T, Bailey SD, Tu WB, et al. BioID identifies novel c-MYC interacting partners in cultured cells and xenograft tumors. Journal of proteomics. 2015;118:95-111.

3. Pedrioli PG. Trans-proteomic pipeline: a pipeline for proteomic analysis. Methods Mol Biol. 2010;604:213-38.

4. Liu G, Zhang J, Larsen B, Stark C, Breitkreutz A, Lin ZY, et al. ProHits: integrated software for mass spectrometry-based interaction proteomics. Nat Biotechnol. 2010;28(10):1015-7.

5. Kessner D, Chambers M, Burke R, Agus D, Mallick P. ProteoWizard: open source software for rapid proteomics tools development. Bioinformatics. 2008;24(21):2534-6.

6. Eng JK, Jahan TA, Hoopmann MR. Comet: an open-source MS/MS sequence database search tool. Proteomics. 2013;13(1):22-4.

7. Lopes CT, Franz M, Kazi F, Donaldson SL, Morris Q, Bader GD. Cytoscape Web: an interactive web-based network browser. Bioinformatics. 2010;26(18):2347-8.

8. Schmitt TM, Zuniga-Pflucker JC. Induction of T cell development from hematopoietic progenitor cells by delta-like-1 in vitro. Immunity. 2002;17(6):749-56.

9. Dobin A, Davis CA, Schlesinger F, Drenkow J, Zaleski C, Jha S, et al. STAR: ultrafast universal RNA-seq aligner. Bioinformatics. 2013;29(1):15-21.

10. Heinz S, Benner C, Spann N, Bertolino E, Lin YC, Laslo P, et al. Simple combinations of lineage-determining transcription factors prime cis-regulatory elements required for macrophage and B cell identities. Mol Cell. 2010;38(4):576-89.

11. Robinson MD, McCarthy DJ, Smyth GK. edgeR: a Bioconductor package for differential expression analysis of digital gene expression data. Bioinformatics. 2010;26(1):139-40.

12. Langmead B, Trapnell C, Pop M, Salzberg SL. Ultrafast and memory-efficient alignment of short DNA sequences to the human genome. Genome biology. 2009;10(3):R25.

13. Li QB JB, Huang H, Bickel PJ. Measuring reproducibility of high-throughput experiments. . Ann Appl Stat 2011;5(5):1752-79.

14. de Hoon MJ, Imoto S, Nolan J, Miyano S. Open source clustering software. Bioinformatics. 2004;20(9):1453-4.

15. Saldanha AJ. Java Treeview--extensible visualization of microarray data. Bioinformatics. 2004;20(17):3246-8.

16. Kent WJ, Sugnet CW, Furey TS, Roskin KM, Pringle TH, Zahler AM, et al. The human genome browser at UCSC. Genome Res. 2002;12(6):996-1006.

17. Huang da W, Sherman BT, Lempicki RA. Systematic and integrative analysis of large gene lists using DAVID bioinformatics resources. Nature protocols. 2009;4(1):44-57.

18. Huang da W, Sherman BT, Lempicki RA. Bioinformatics enrichment tools: paths toward the comprehensive functional analysis of large gene lists. Nucleic Acids Res. 2009;37(1):1-13.

19. Jensen CT, Ahsberg J, Sommarin MNE, Strid T, Somasundaram R, Okuyama K, et al. Dissection of progenitor compartments resolves developmental trajectories in B-lymphopoiesis. J Exp Med. 2018.

20. Buenrostro JD, Giresi PG, Zaba LC, Chang HY, Greenleaf WJ. Transposition of native chromatin for fast and sensitive epigenomic profiling of open chromatin, DNA-binding proteins and nucleosome position. Nat Methods. 2013;10(12):1213-8.

21. Langmead B, Salzberg SL. Fast gapped-read alignment with Bowtie 2. Nat Methods. 2012;9(4):357-9.

22. Quinlan AR, Hall IM. BEDTools: a flexible suite of utilities for comparing genomic features. Bioinformatics. 2010;26(6):841-2.

23. Zhou X, Maricque B, Xie M, Li D, Sundaram V, Martin EA, et al. The Human Epigenome Browser at Washington University. Nat Methods. 2011;8(12):989-90.

24. Fang R, Yu M, Li G, Chee S, Liu T, Schmitt AD, et al. Mapping of long-range chromatin interactions by proximity ligation-assisted ChIP-seq. Cell Res. 2016;26(12):1345-8.

25. Servant N, Varoquaux N, Lajoie BR, Viara E, Chen CJ, Vert JP, et al. HiC-Pro: an optimized and flexible pipeline for Hi-C data processing. Genome biology. 2015;16:259.

26. Li H, Handsaker B, Wysoker A, Fennell T, Ruan J, Homer N, et al. The Sequence Alignment/Map format and SAMtools. Bioinformatics. 2009;25(16):2078-9.

27. Conway JR, Lex A, Gehlenborg N. UpSetR: an R package for the visualization of intersecting sets and their properties. Bioinformatics. 2017;33(18):2938-40.

28. Gu Z, Gu L, Eils R, Schlesner M, Brors B. circlize Implements and enhances circular visualization in R. Bioinformatics. 2014;30(19):2811-2.

29. Lilljebjorn H, Henningsson R, Hyrenius-Wittsten A, Olsson L, Orsmark-Pietras C, von Palffy S, et al. Identification of ETV6-RUNX1-like and DUX4-rearranged subtypes in paediatric B-cell precursor acute lymphoblastic leukaemia. Nature communications. 2016;7:11790.

30. Gu Z, Churchman ML, Roberts KG, Moore I, Zhou X, Nakitandwe J, et al. PAX5-driven subtypes of B-progenitor acute lymphoblastic leukemia. Nat Genet. 2019.

31. Love MI, Huber W, Anders S. Moderated estimation of fold change and dispersion for RNA-seq data with DESeq2. Genome biology. 2014;15(12):550.

32. Liu GJ, Cimmino L, Jude JG, Hu Y, Witkowski MT, McKenzie MD, et al. Pax5 loss imposes a reversible differentiation block in B-progenitor acute lymphoblastic leukemia. Genes Dev. 2014;28(12):1337-50.
