# Supplementary figures and images for: PAX5 is part of a functional transcription factor network targeted in lymphoid leukemia
Source: PLoS Genet. 2019 Aug 5;15(8):e1008280. doi: 10.1371/journal.pgen.1008280 (PMC6695195; doi:10.1371/journal.pgen.1008280)

# Figure S1

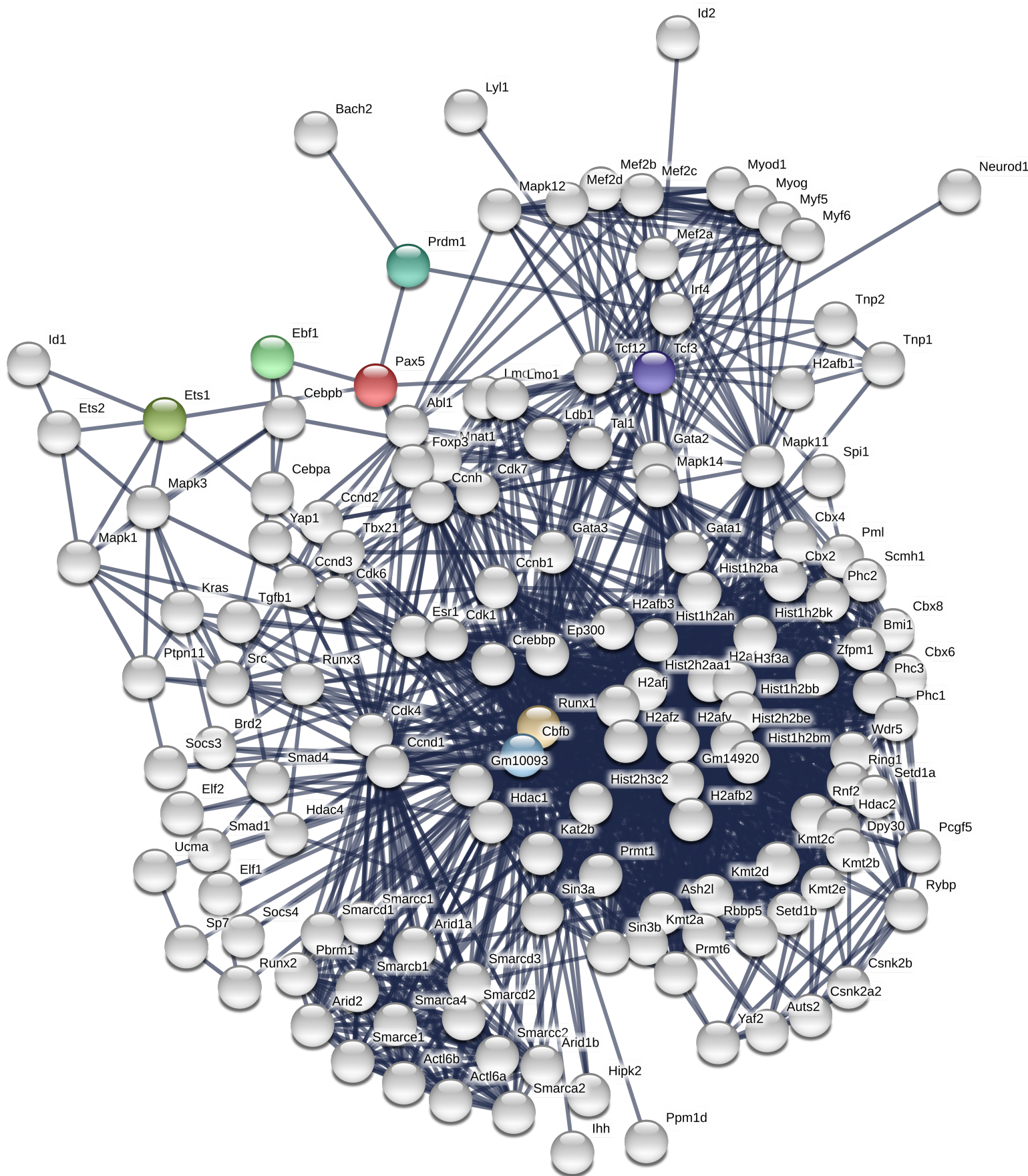

Supplement: S1 Fig — The figure displays a network map generated by analysis of the PAX5 interactome using Search Tool for the Retrieval of Interacting Genes/Proteins (STRING) (https://string-db.org). Colored nodes indicate the query protein (PAX5, Red) and first shell of interactors. Meanwhile, the light grey nodes indicate the second shell of interactors. Edges represent protein-protein associations. The minimum required interaction score was 0.9. (PDF) [file pgen.1008280.s011.pdf]

Figure S2

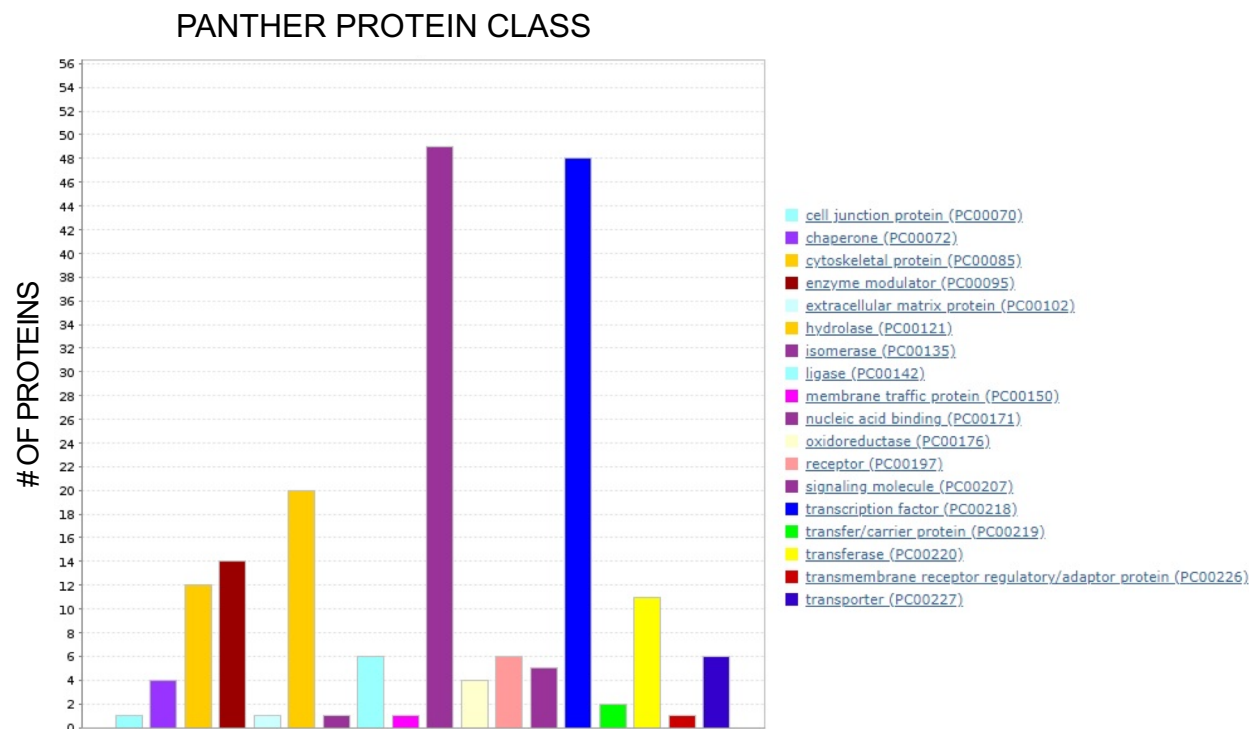

Supplement: S2 Fig — The diagram displays a functional enrichment analysis identified overrepresented protein classes in the dataset by Gene ontology (GO) analysis performed with PANTHER14.0. (PDF) [file pgen.1008280.s012.pdf]

# Figure S3 Fraction of Pax5 PXI mutated Tumors vs Total data set

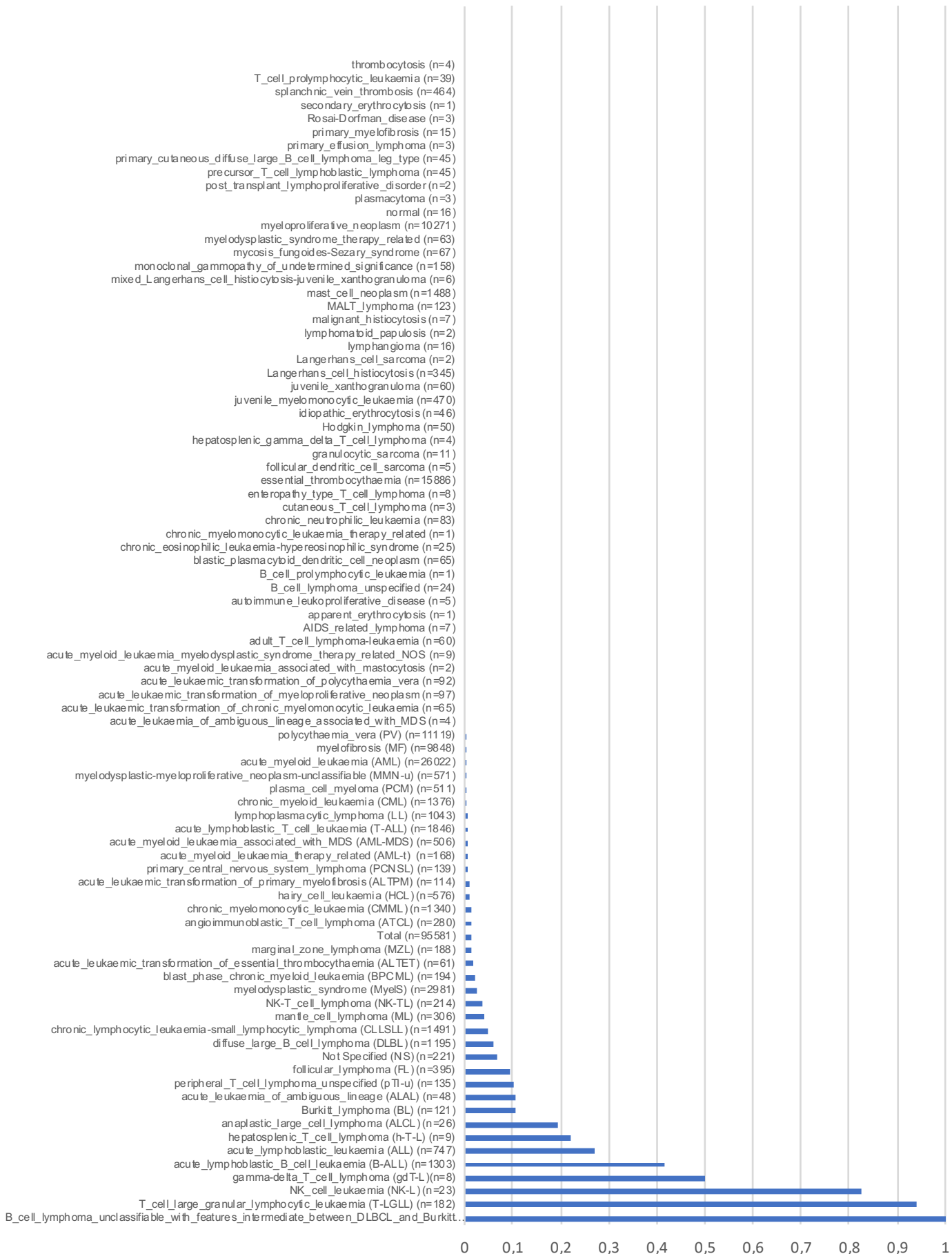

Supplement: S3 Fig — The diagram displays the fraction of tumors of different histological origin with reported PAX5 PXI mutations vs total number of reported tumors per type are listed. The analysis was based on a list of 239 PAX5 PXI´s that were investigated in the public cancer database COSMIC (cancer genes sensus V76) obtained from the Sanger Institute Catalogue Of Somatic Mutations In Cancer web site, (http://cancer.sanger.ac.uk/cosmic) [14]. Only entries classified as hematopoietic and lymphoid tissue are considered. (PDF) [file pgen.1008280.s013.pdf]

Figure S7

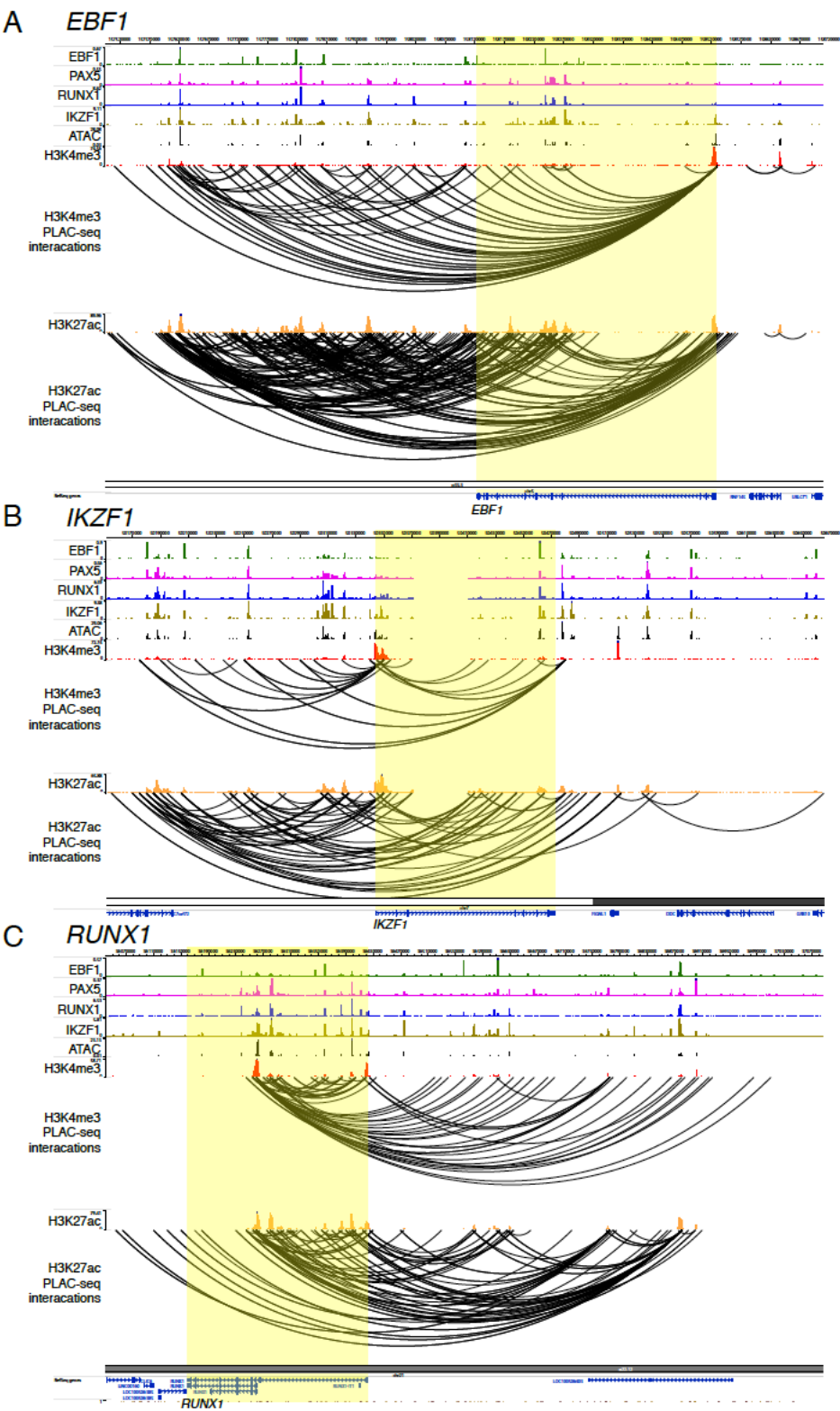

Supplement: S7 Fig — The figure panels (A-C) displays WashU Genome Browser tracks of the EBF1, IKZF1 and RUNX1 genes in NALM6 B-leukemic cells. ChIP-seq data of EBF1, PAX5, RUNX1 and IKZF1 were linked to H3K4me3, H3K27ac chromatin marks and ATAC-seq as well as H3K4me3 and H3K27ac anchored PLAC-seq interactions on the EBF1 (A) IKZF1 (B) and RUNX1 (C) genes. The data is visualized in the WashU Genome Browser. (PDF) [file pgen.1008280.s017.pdf]

Figure S8

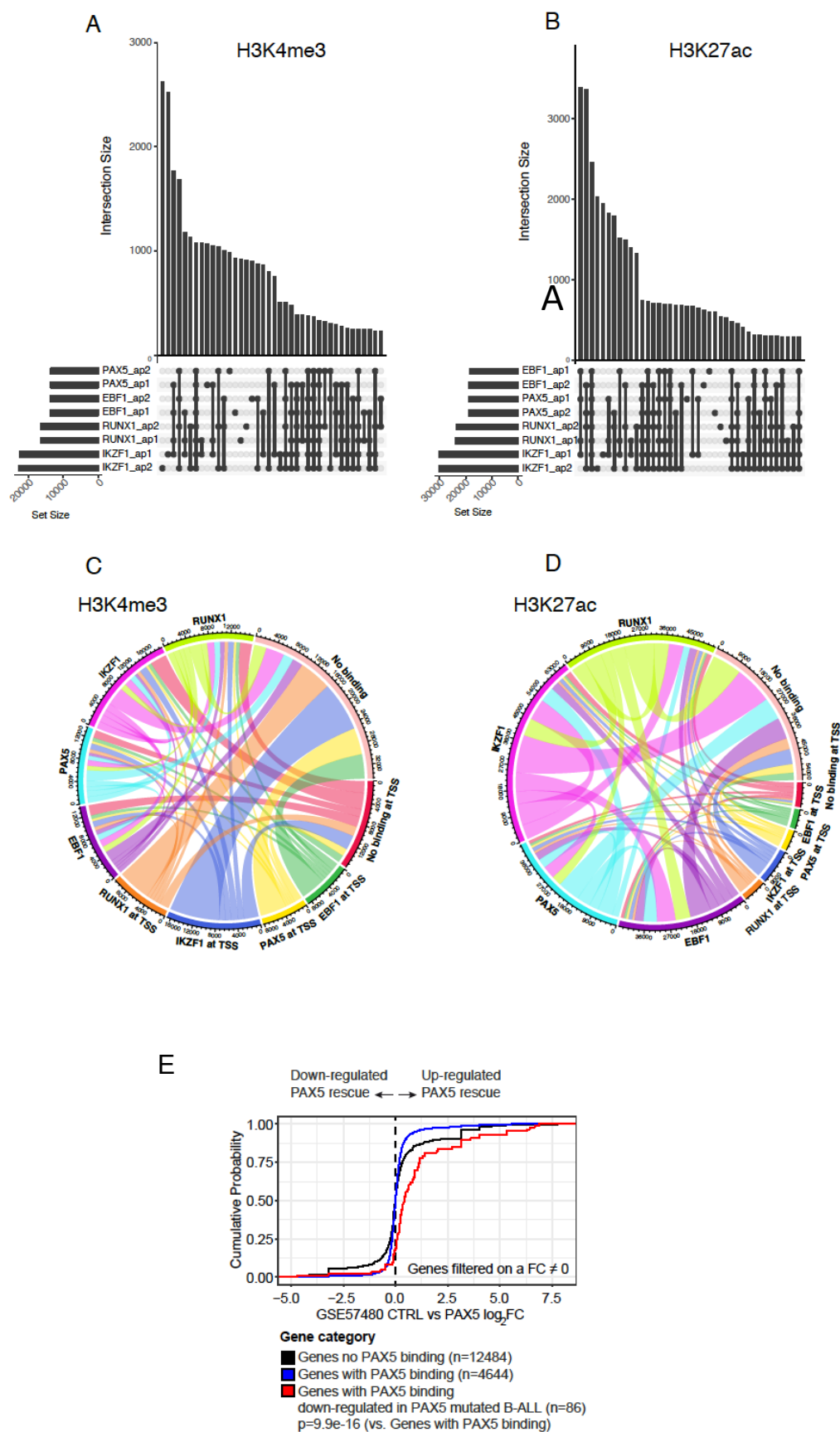

Supplement: S8 Fig — Panel (A, B) show diagrams displaying the abundance of combined PAX5, EBF1, RUNX1 and/or IKZF1 binding at identified PLAC-seq anchor-points. Top 40 combinations out of 251 possible for H3K27ac (A) and 252 for anchor-point bound H3K4me3 (B) PLAC-seq interactions are visualized as UpSet plots. Interactions were filtered for PAX5, EBF1, RUNX1 and/or IKZF1 overlapping ChIP-seq peaks in either one or both anchor-points (for details, see Methods). UpSet plots describe how the combination of TFs in one anchor-point (ap) relates to combination of TFs in the other. Panel (C-D) show Chord diagram displaying distal-to-TSS (transcription start site) anchored PAX5-EBF1-RUNX1-IKZF1 gene networks in NALM6 cells. C) H3K4me3 or D) H3K27ac anchored PLAC-seq was used to define chromatin interactions in NALM6 cells. Interactions were filtered for PAX5, EBF1, RUNX1 and/or IKZF1 overlapping ChIP-seq peaks in either one or both anchor-points Anchor points were defined as either distal (more than 2.5kb away from TSS) or as TSS-anchored (within 2.5kb from TSS). The chord diagrams were visualized with the circlize package in R. (E) ECDF plot displaying the association of PAX5 binding in NALM6 cells and gene expression changes upon introduction of PAX5 in PAX5 mutated REH cells (GSE57480). The following gene categories are shown: Red line describes genes identified as down regulated in patient samples carrying a mutated PAX5 gene and targeted for PAX5 binding in NALM6 cells (Fig 5A, S8 Table). Blue line shows all genes with PAX5 binding in NALM6 independent of their gene expression status in primary human B-ALL samples (genes in the differentially expressed (red) category were excluded). Black line describes the expression changes of PAX5 unbound genes upon PAX5 reintroduction. Kolmogorov-Smirnov p-values are shown. (PDF) [file pgen.1008280.s018.pdf]
