# Supplementary material for: PAX5 is part of a functional transcription factor network targeted in lymphoid leukemia
Source: PLoS Genet. 2019 Aug 5;15(8):e1008280. doi: 10.1371/journal.pgen.1008280 (PMC6695195; doi:10.1371/journal.pgen.1008280)

FgS4

A.

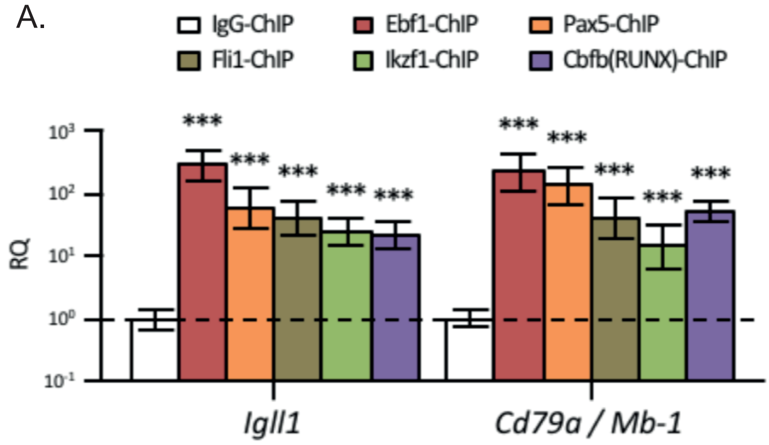

B.

	Rank	*De novo* Motif	P-value	% T/Bg	Best match
230-238 PAX5 peaks	1.	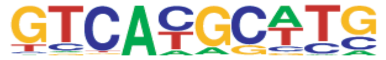	1e-463	18.5/5.7	PAX1
	2.	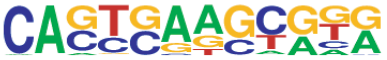	1e-437	16.7/5.0	PAX8
	3.	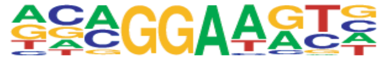	1e-235	33.7/20.2	ETV2 (ETS)
	9.	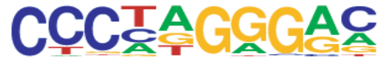	1e-110	15.9/9.1	EBF
	11.	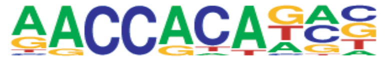	1e-83	14.5/8.8	RUNX2
230-238 IKZF1 peaks	1.	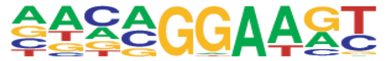	1e-318	36.7/19.4	ETV2 (ETS)
	2.	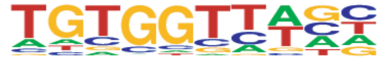	1e-183	10.3/3.5	RUNX
	3.	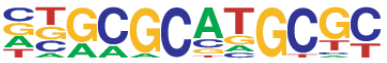	1e-119	6.9/2.4	NRF1
	5.	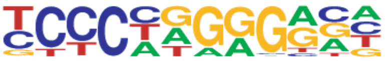	1e-101	17.9/10.4	EBF
230-238 RUNX1 peaks	1.	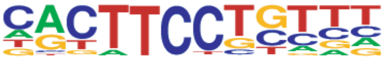	1e-1403	29.5/9.9	ETS1 (ETS)
	2.	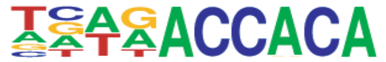	1e-1055	17.2/4.5	RUNX2
	3.	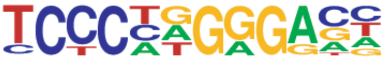	1e-525	10.9/3.4	EBF

C.

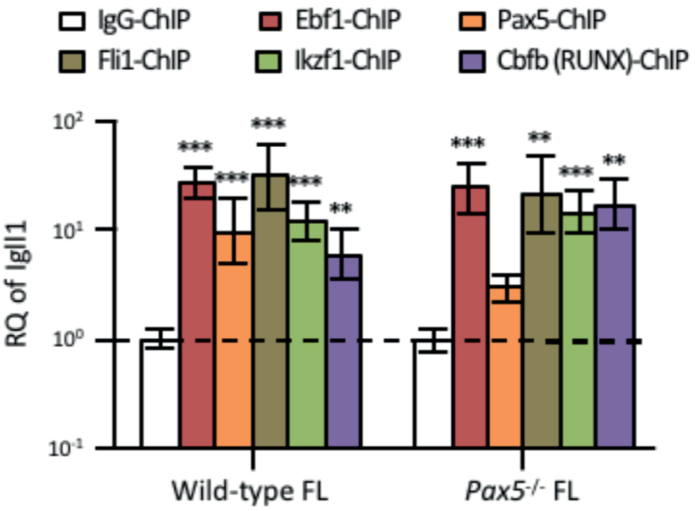

D.

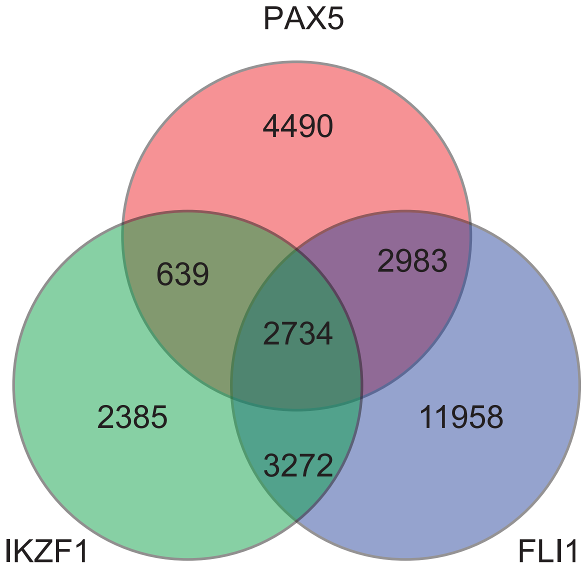

Supplement: S4 Fig — (A) Diagrams displaying Q-PCR data for co-precipitations of the Igll1 and Cd79a/Mb-1 promoters after ChIP of EBF1 (n = 7), PAX5 (n = 3), FLI1 (n = 4), IKZF1 (n = 4), and CBF0β (n = 3) from 230–238 Pre-B cells. The diagrams are based on the relative enrichment as compared to a polyclonal IgG. Statistical analysis was based on Student’s t-tests (two-tailed). ***: p < 0.0005 (B) Peak files from ChIP-seq data of PAX5, IKZF1 and RUNX1 in 230–238 cells (Fig 3A) were analyzed for motif enrichment using findMotifsGenome.pl in Homer (mm10 -size 200). Rank, enriched motif, P-value, % of target (T) and background (Bg) and best match to known motifs of Top 3 motifs plus PAX5, EBF1 and RUNX1 motif when present for each peak set are listed. (C) Diagrams displaying Q-PCR data for co-precipitations of Igll1 promoter after ChIP-experiments in Wt, or Pax5-/- pro-B cells. EBF1 (n = 5), PAX5 (n = 3), FLI1 (n = 3), IKZF1 (n = 5), and CBFβ (n = 3). P-Values were based on Student’s t-test (two-tailed) ***: p < 0.0005, **: p < 0.005, *: p < 0.05 RQ: relative quantity. Panel (D) display a Venn-diagram of ChIP-seq peaks based on PAX5, IKZF1 and FLI1 ChIP-seq analysis using the 230–238 Pre-B cell line. Peaks were called using the HOMER platform (findPeaks -style factor) and resulting files were filtered for peaks ≥15 normalized tags. Overlapping peaks were identified using the mergePeaks command in HOMER. (PDF) [file pgen.1008280.s014.pdf]
