# Supplementary material for: PAX5 is part of a functional transcription factor network targeted in lymphoid leukemia
Source: PLoS Genet. 2019 Aug 5;15(8):e1008280. doi: 10.1371/journal.pgen.1008280 (PMC6695195; doi:10.1371/journal.pgen.1008280)

Figure S5A

Pax5 expression

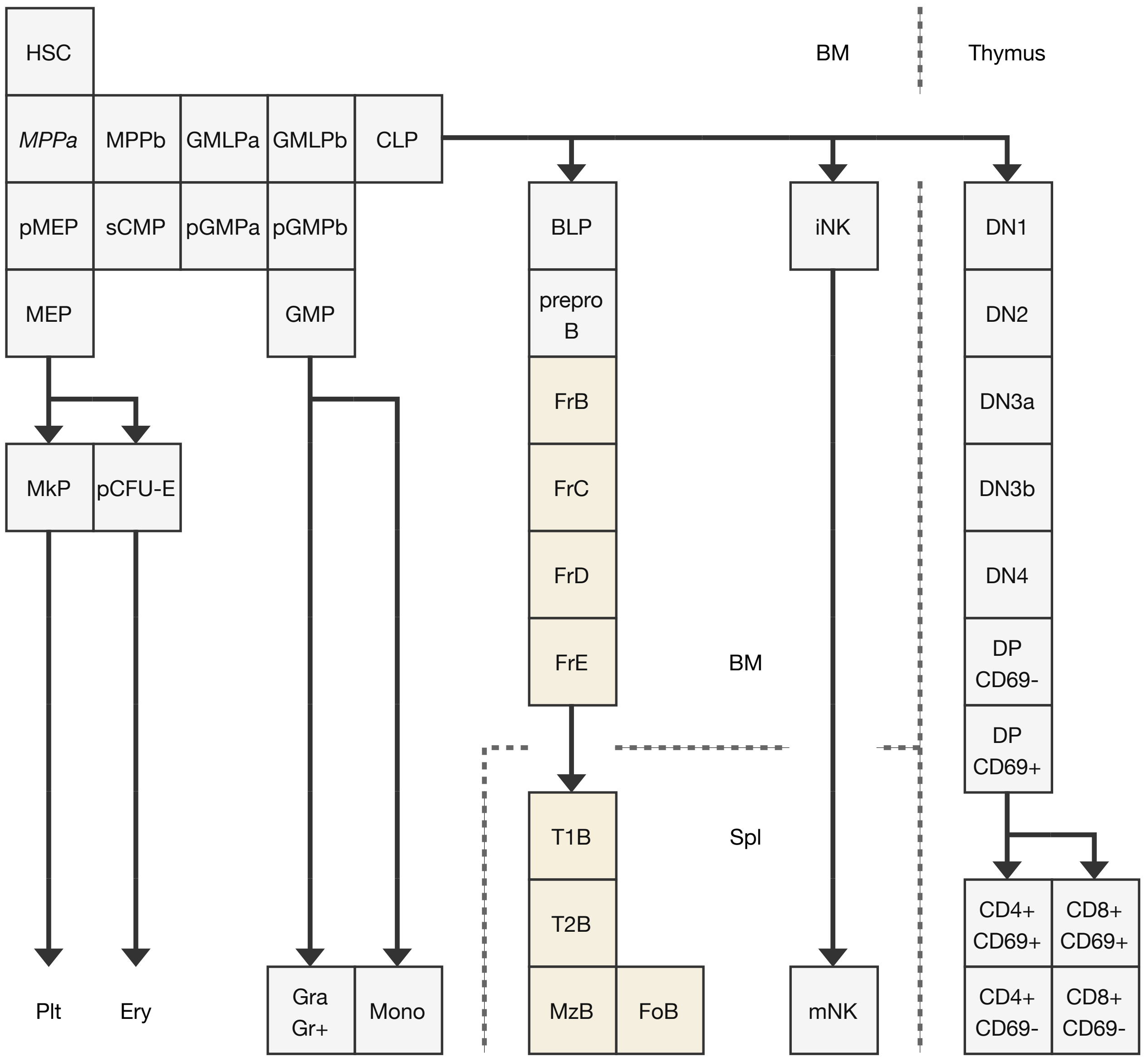

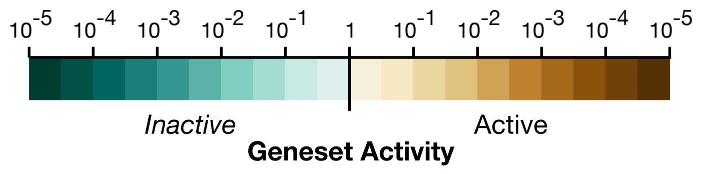

IKZF1 expression

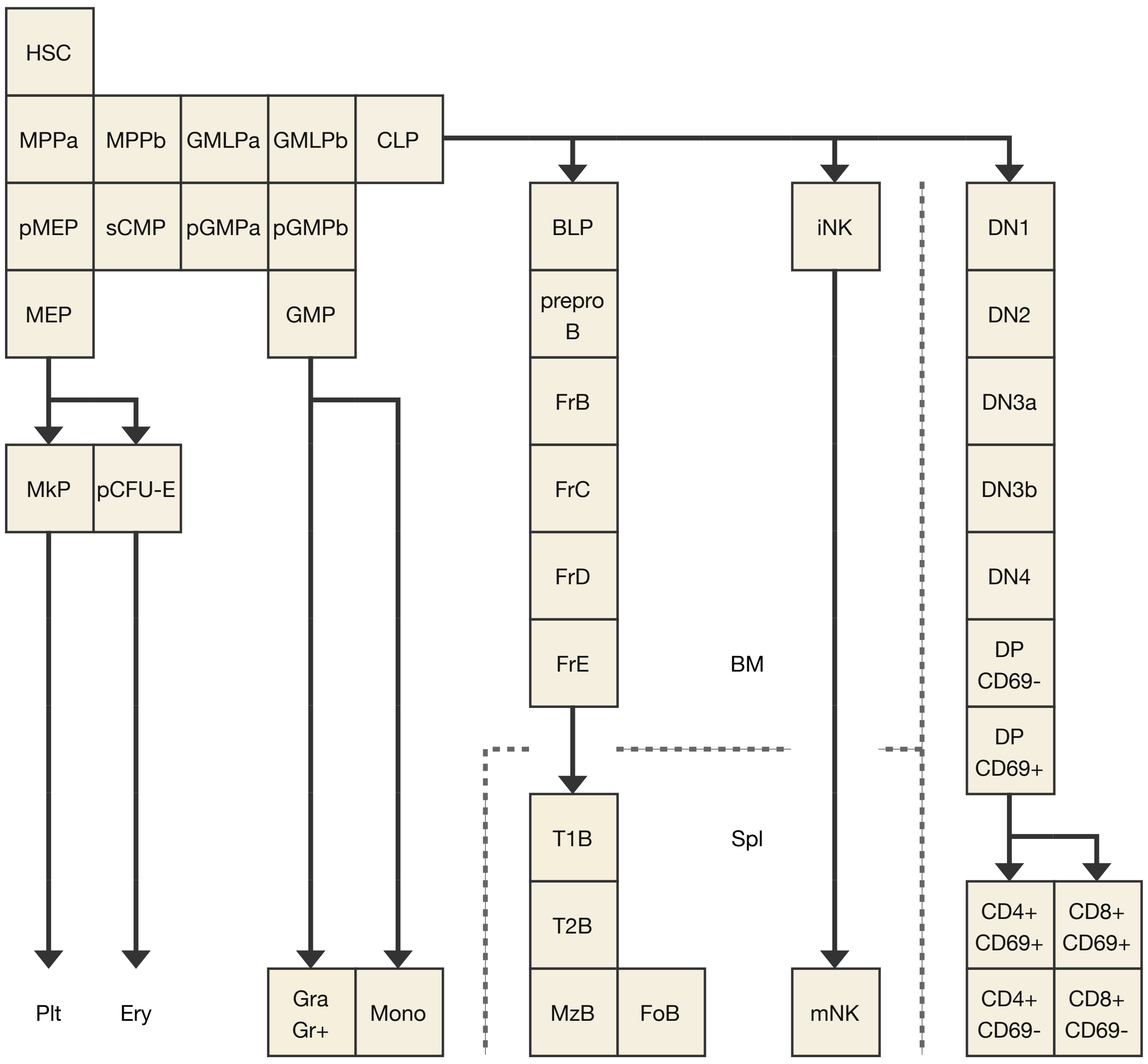

Runx1 expression

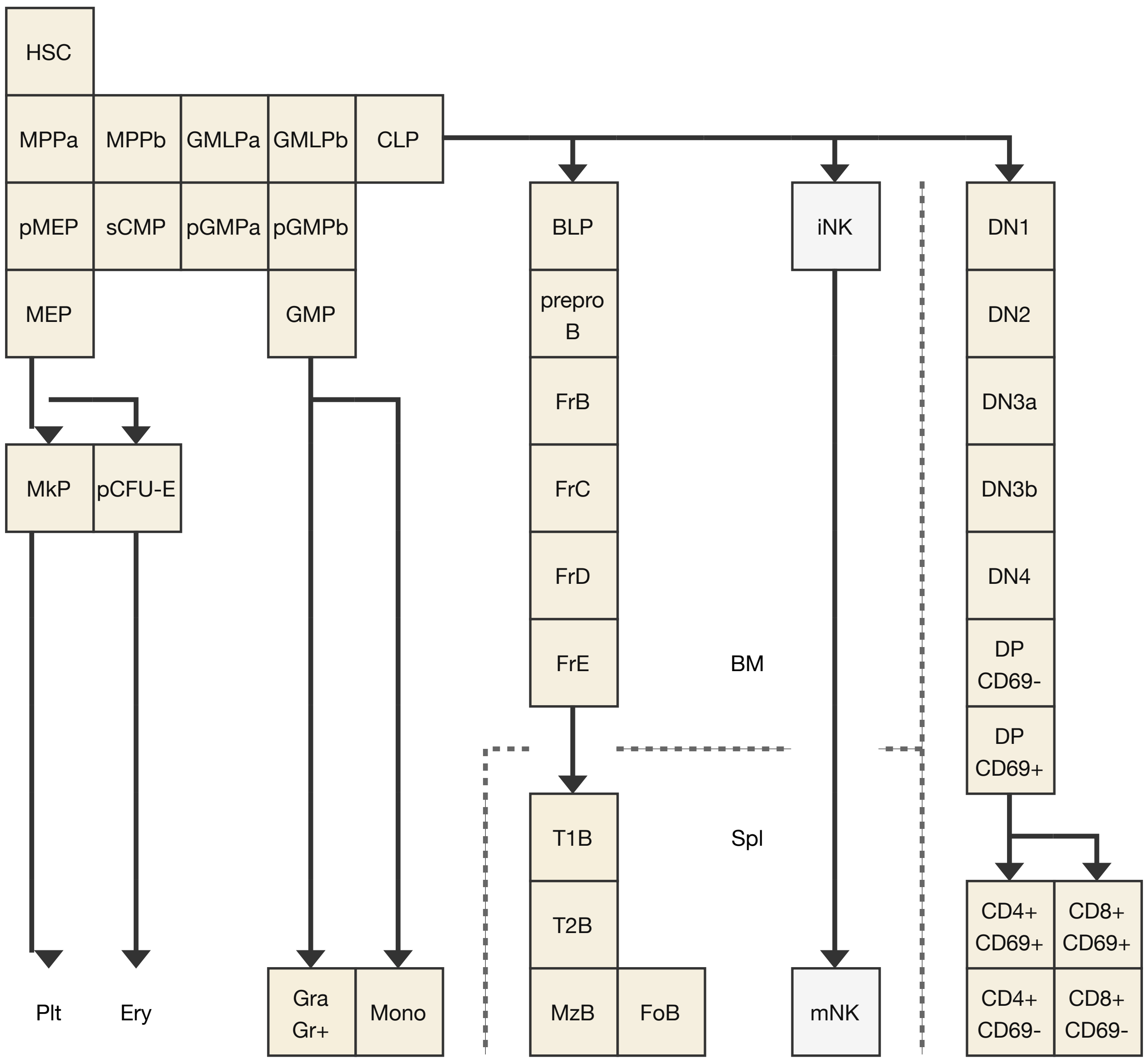

Figure S5B

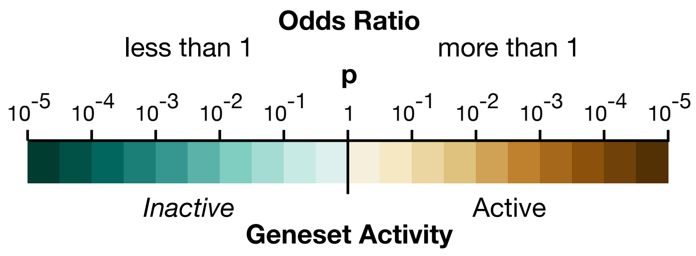

PAX5 targets B-cells geneset

Blk, Ebf, CD19, VpreB1, Igll1, CD79a

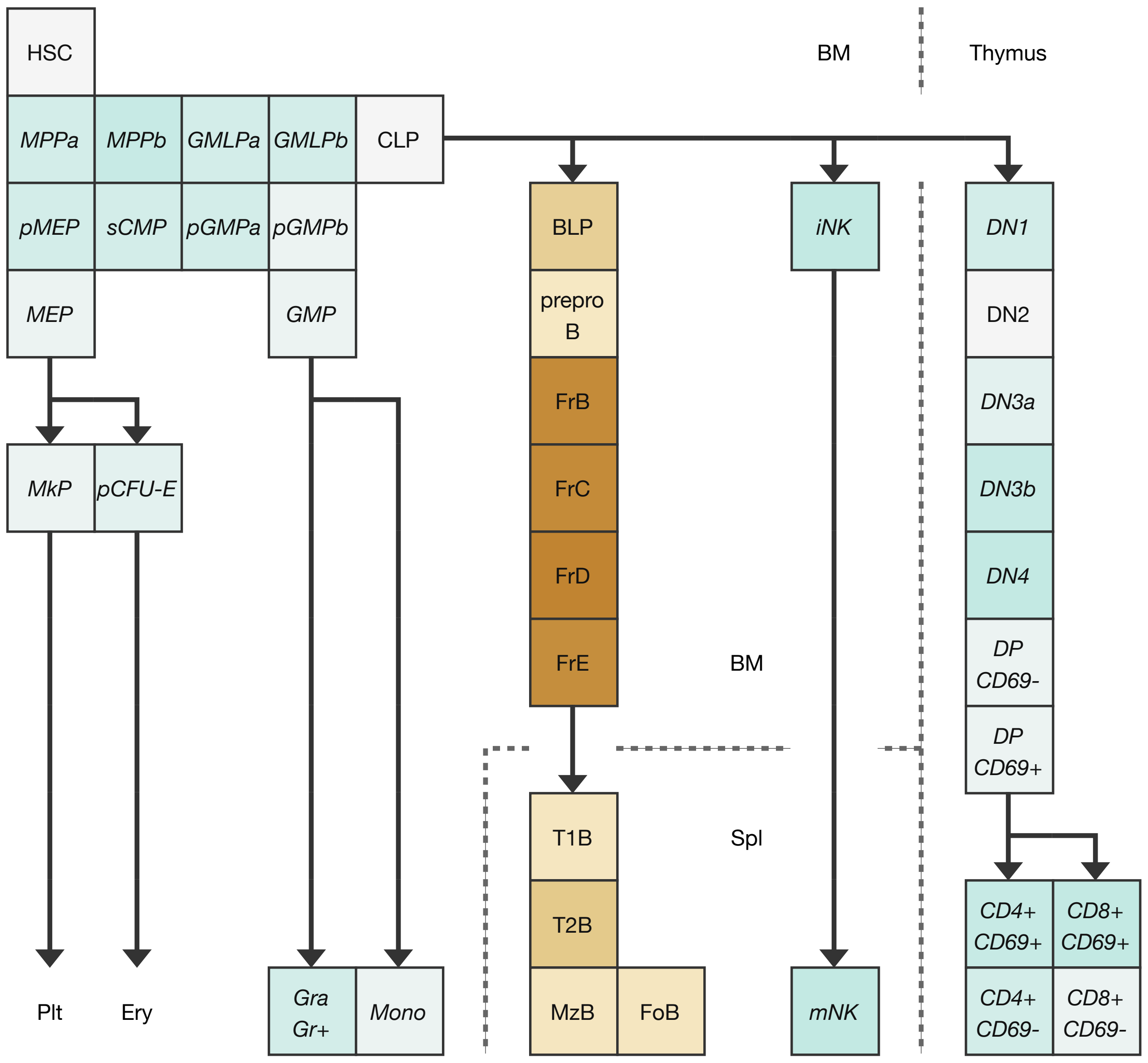

Pax5 unique sites geneset

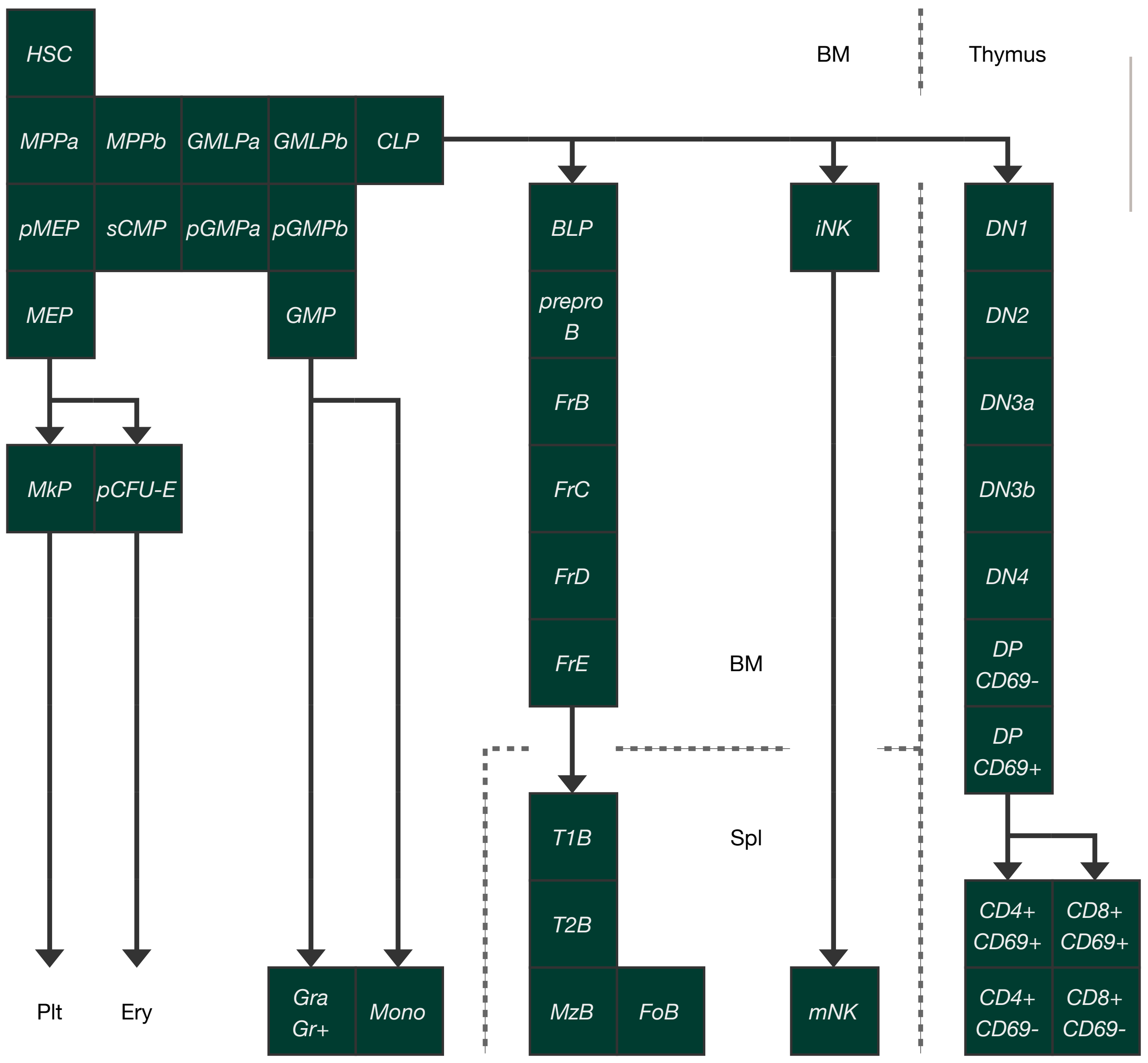

Runx1 unique sites geneset

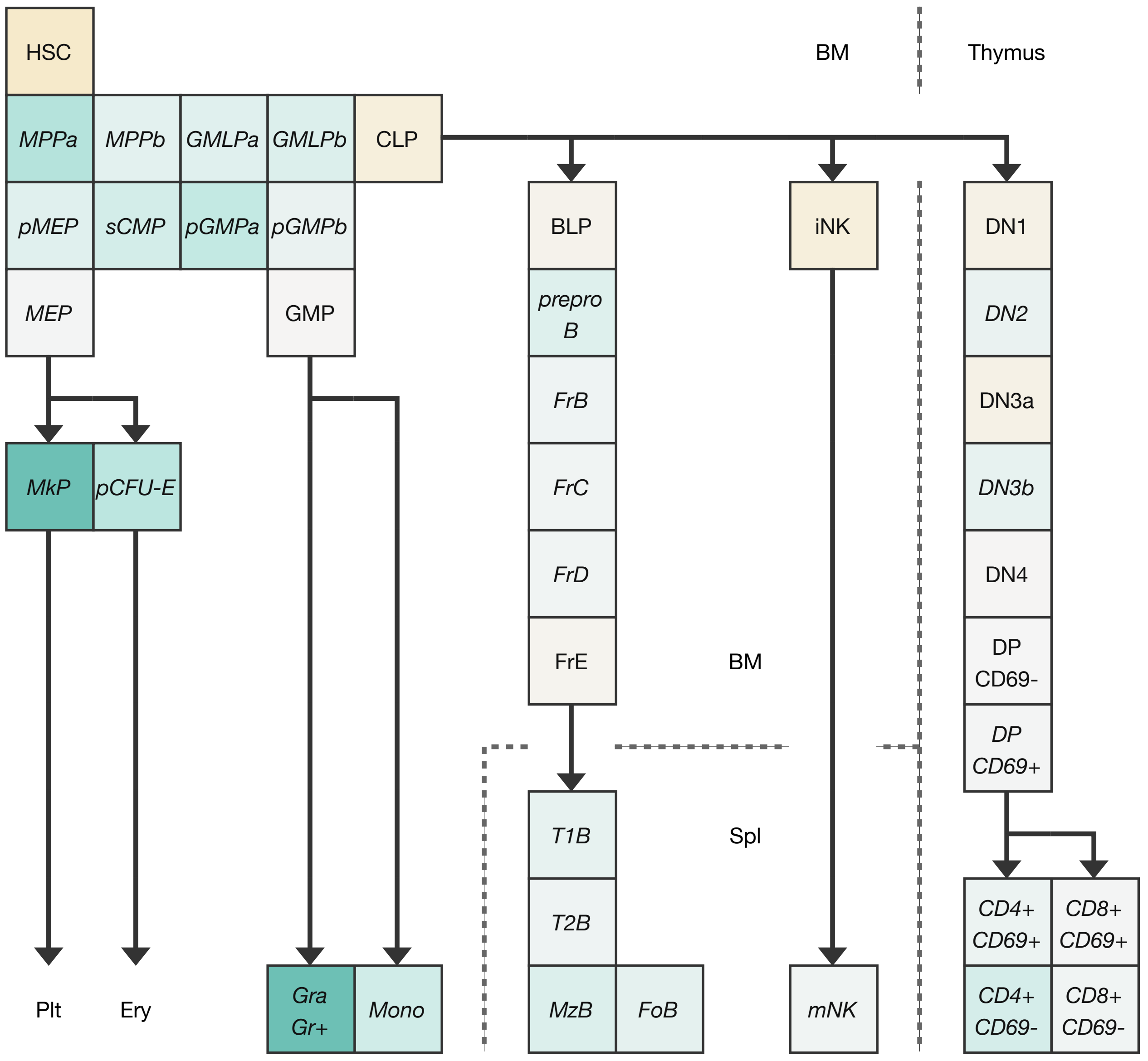

[illegible] unique [illegible] geneset

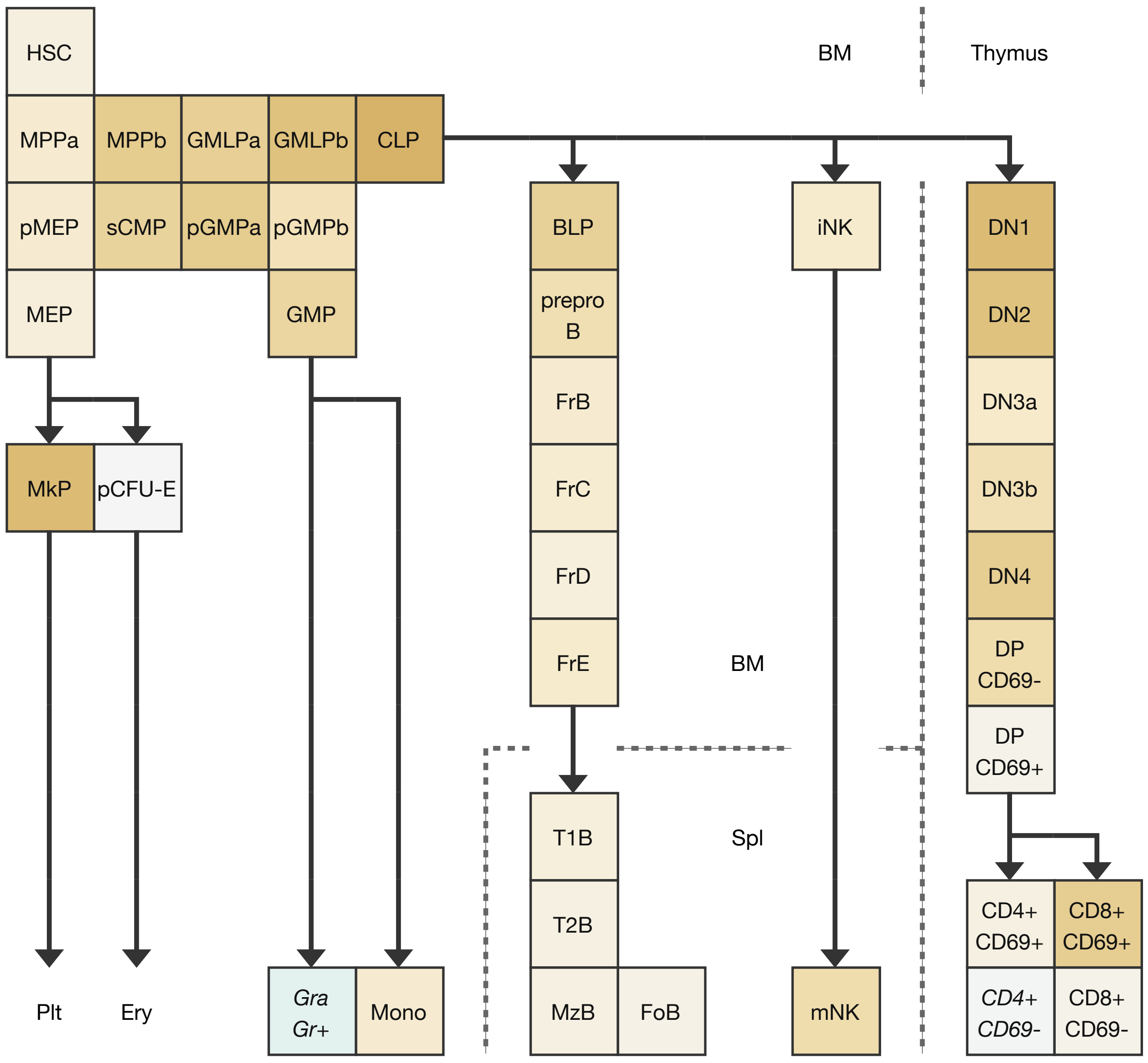

Share this result

Figure S5C

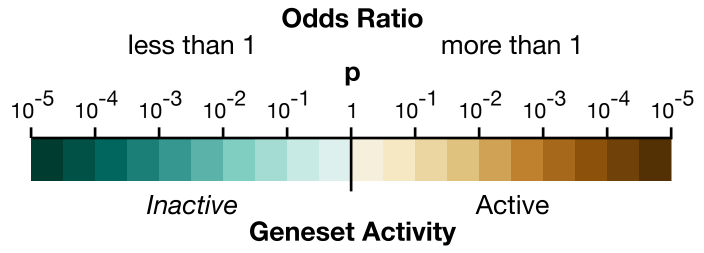

Pax5/Runx unique sites geneset

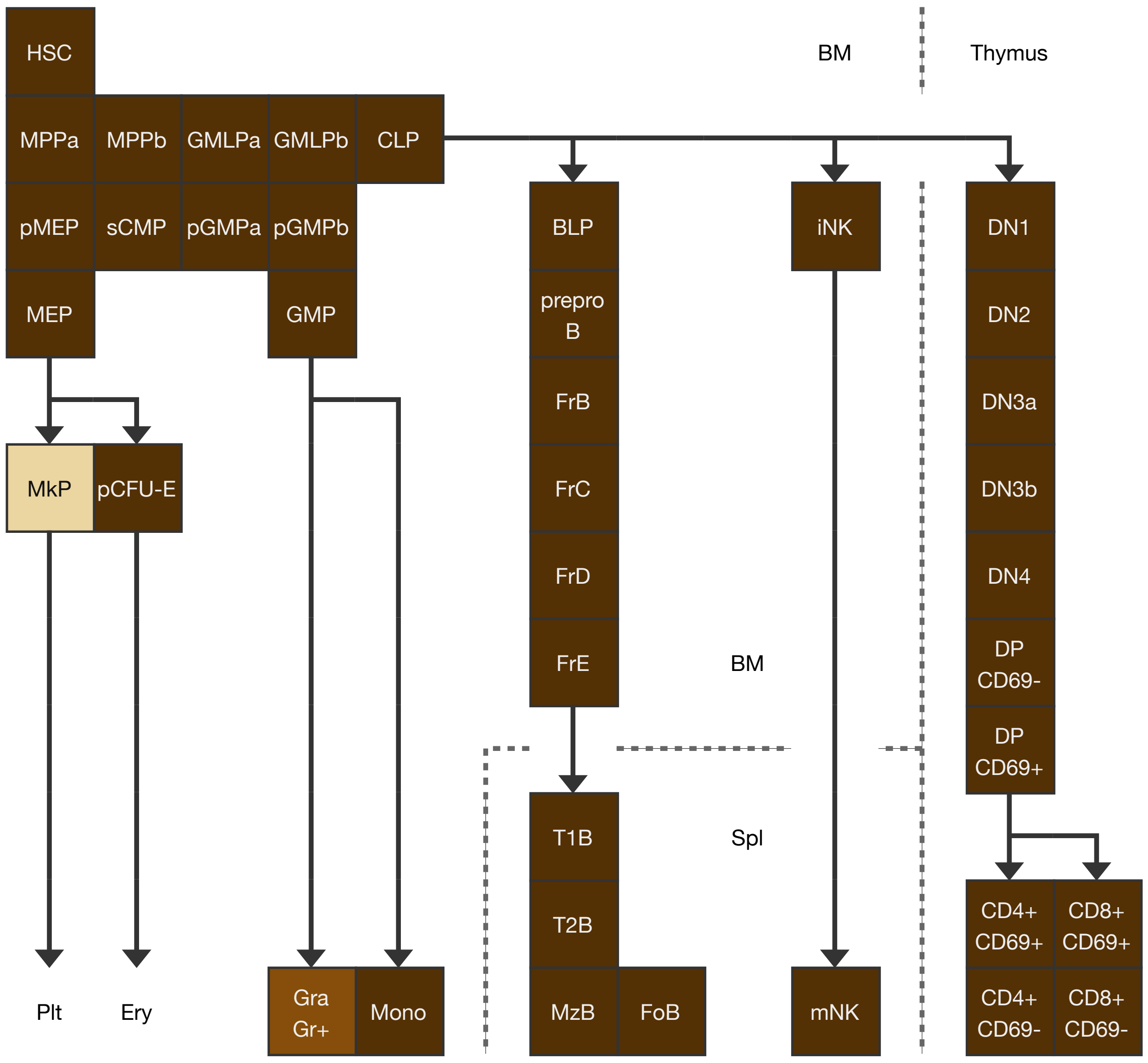

Pe|53Fpm5 yrmuyi wmxiw geneset

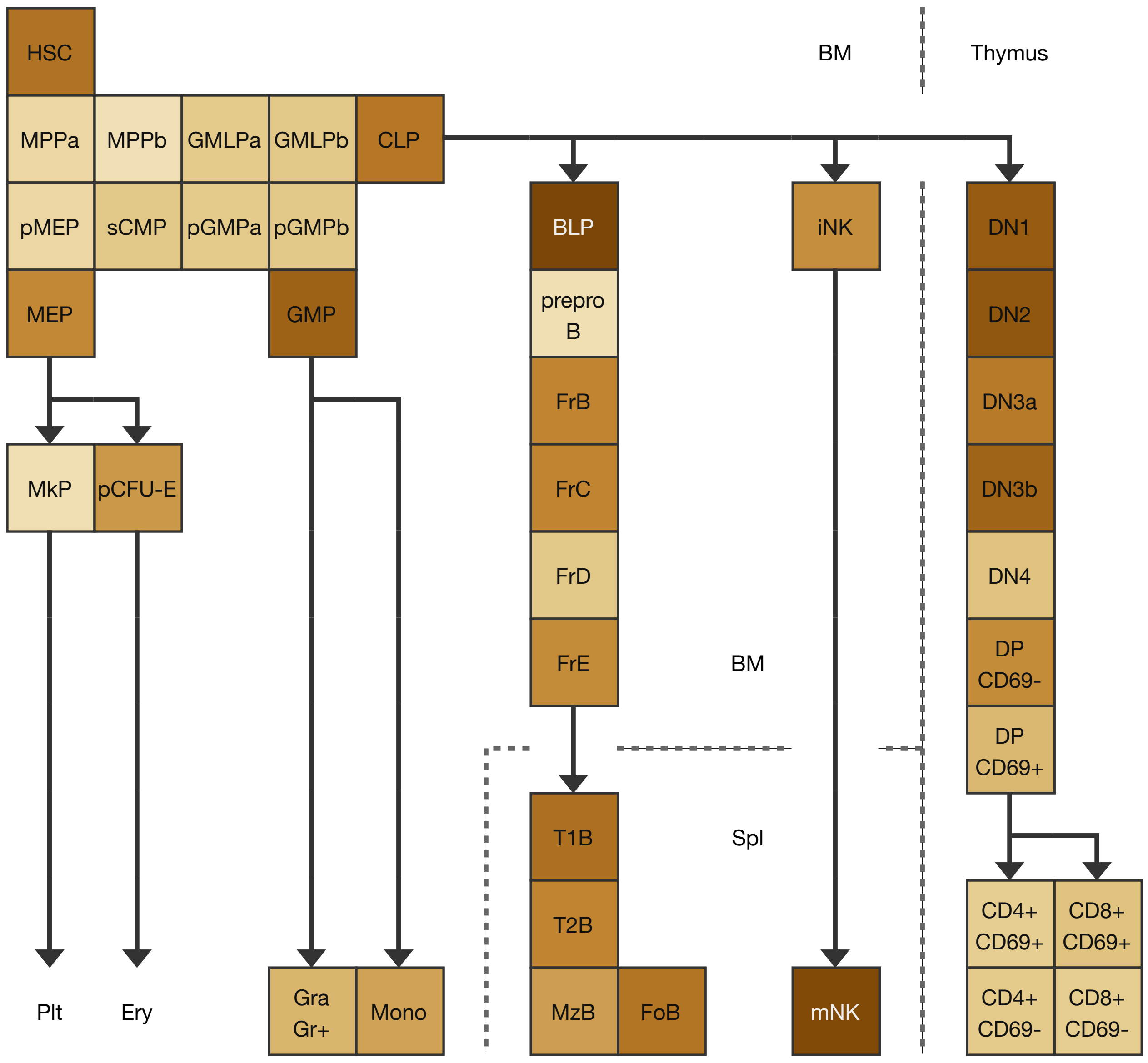

Pax5/Runx1/Ikzf1 unique sites geneset

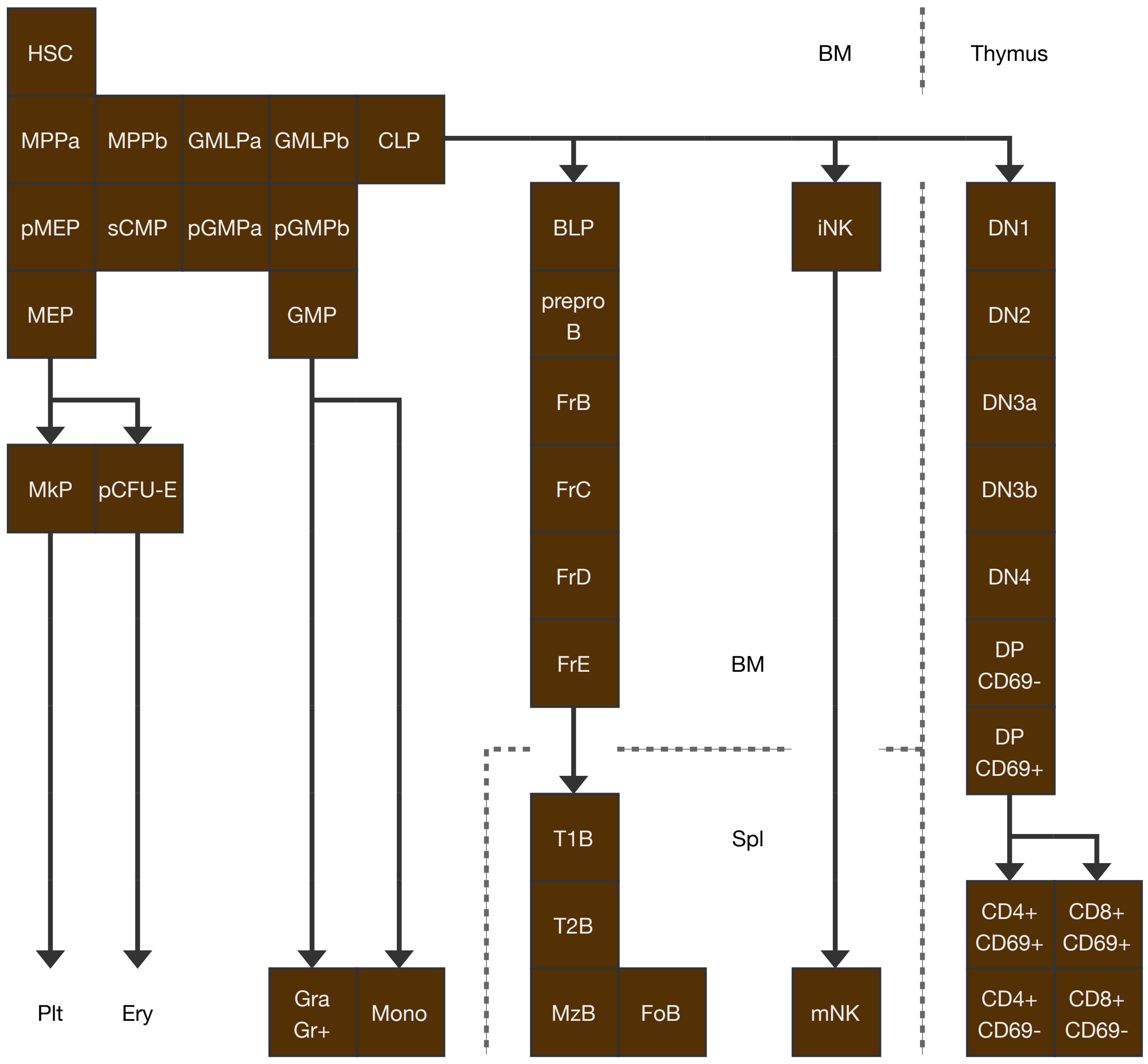

Runx1/Ikzf1 unique geneset

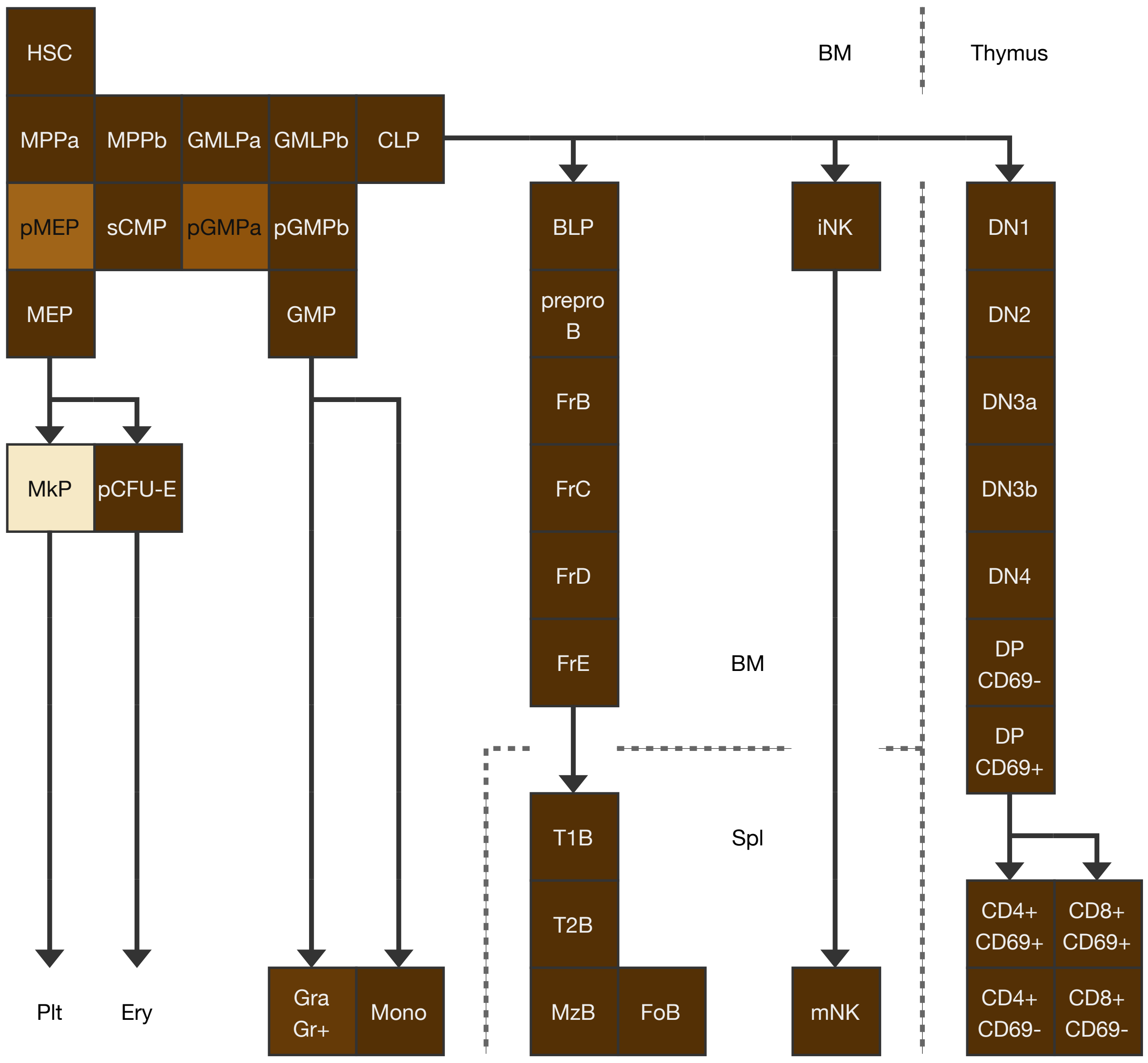

Figure S5D

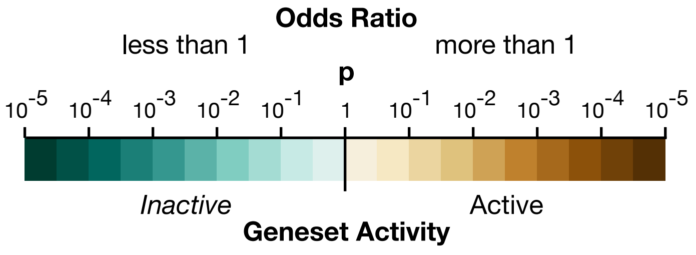

Fli1 expression

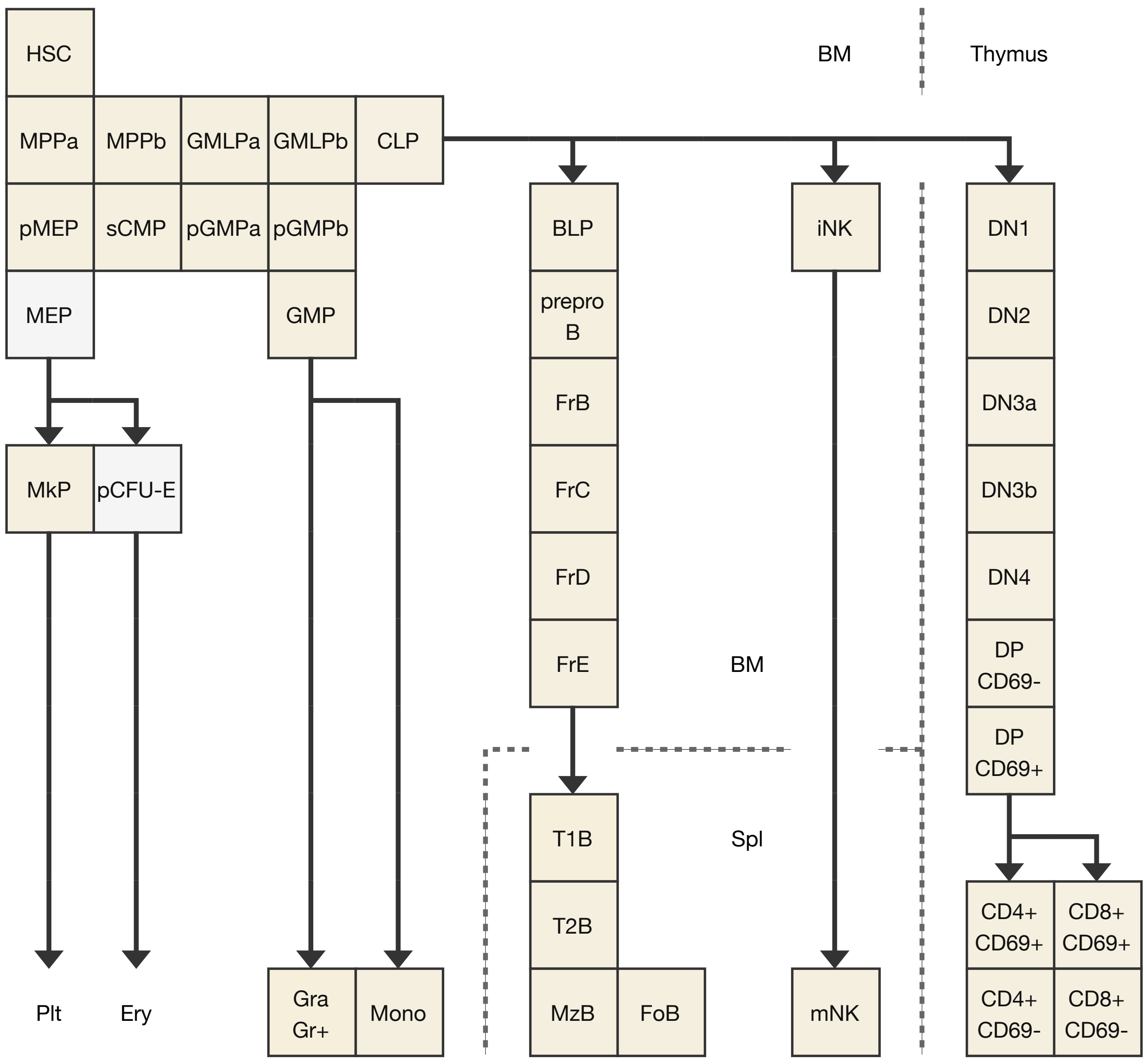

Fli1 unique sites geneset

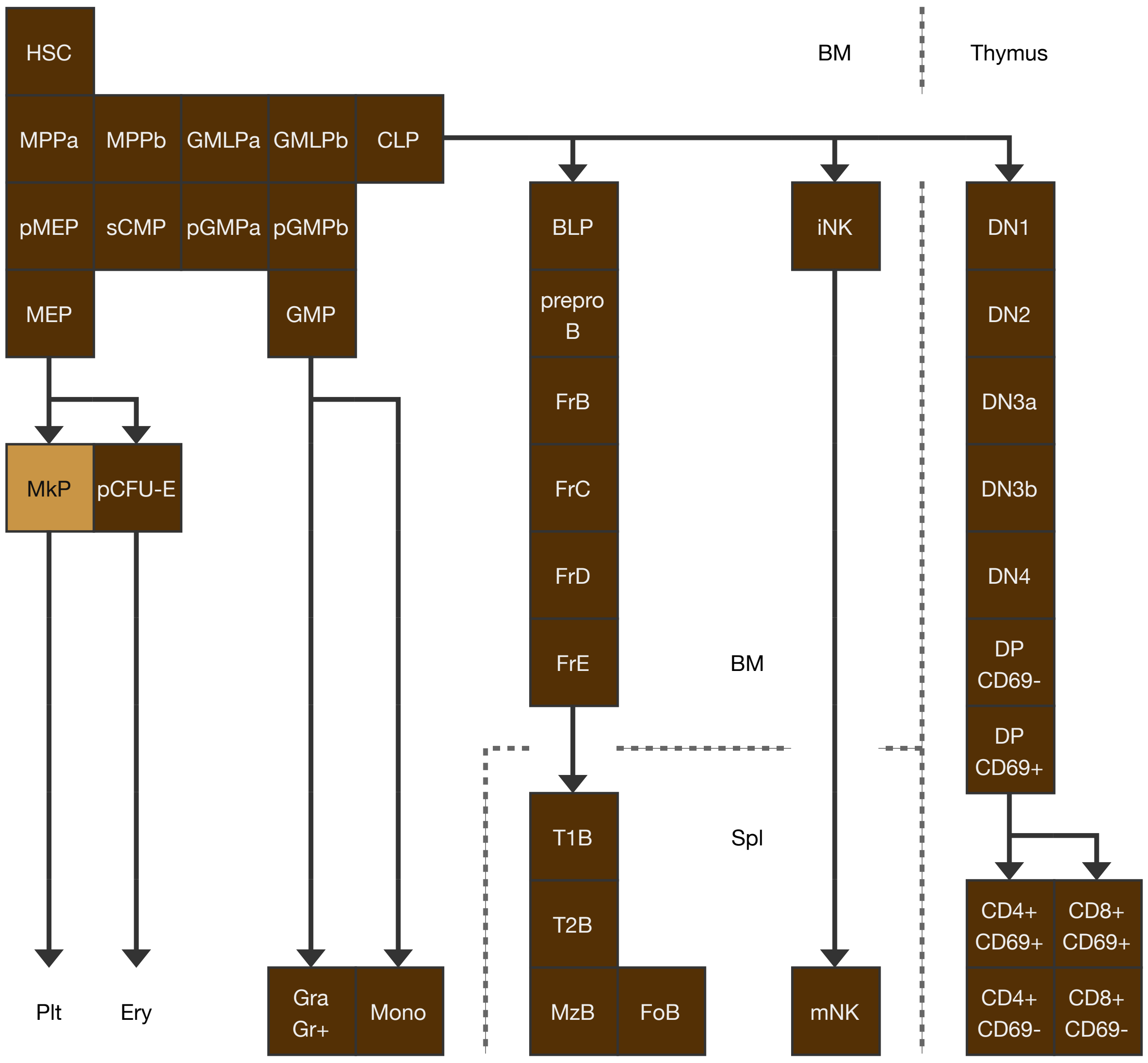

Mo~j5 yrmuyi wmxi geneset

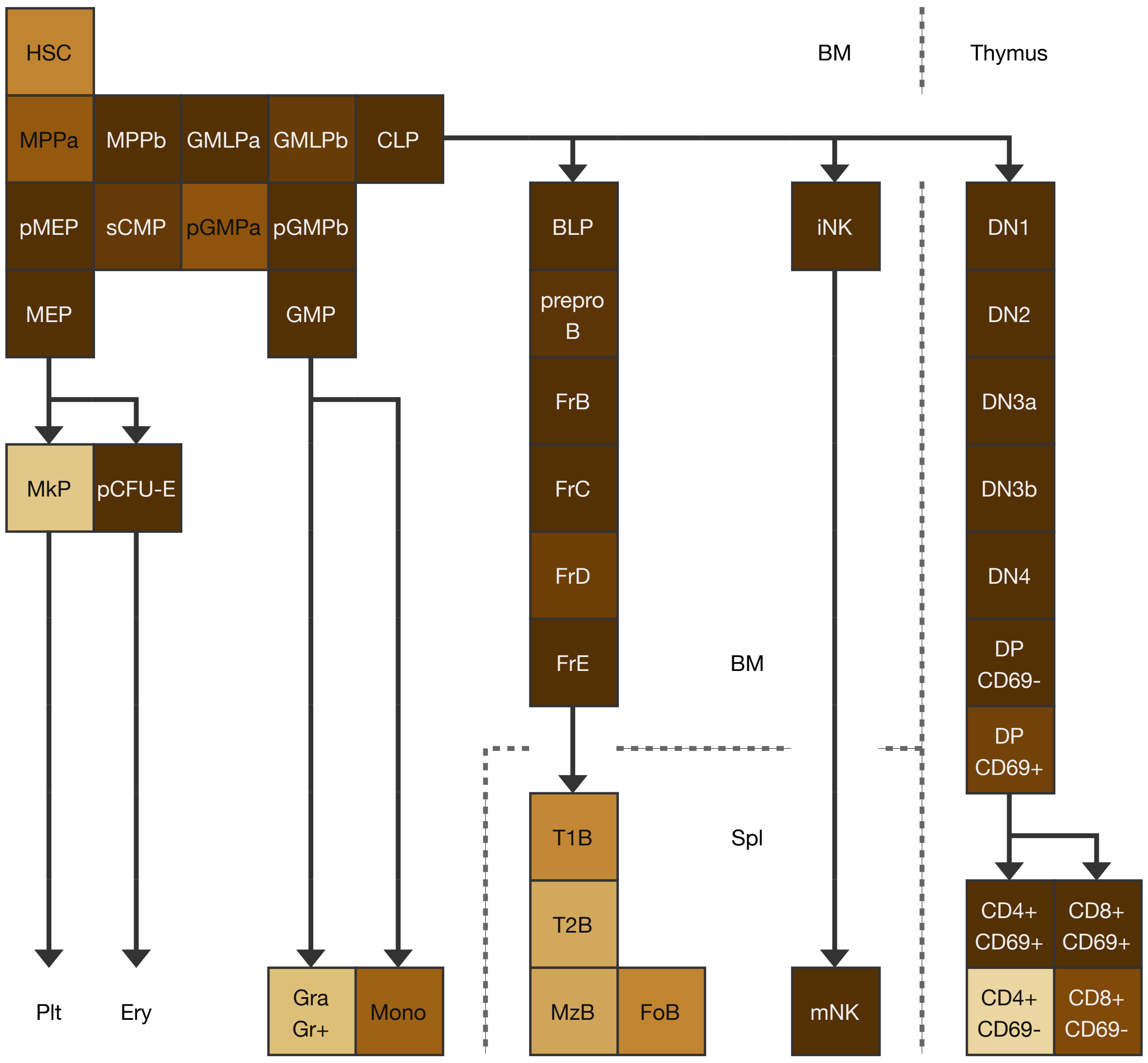

Pax5/Fli1 unique sites geneset

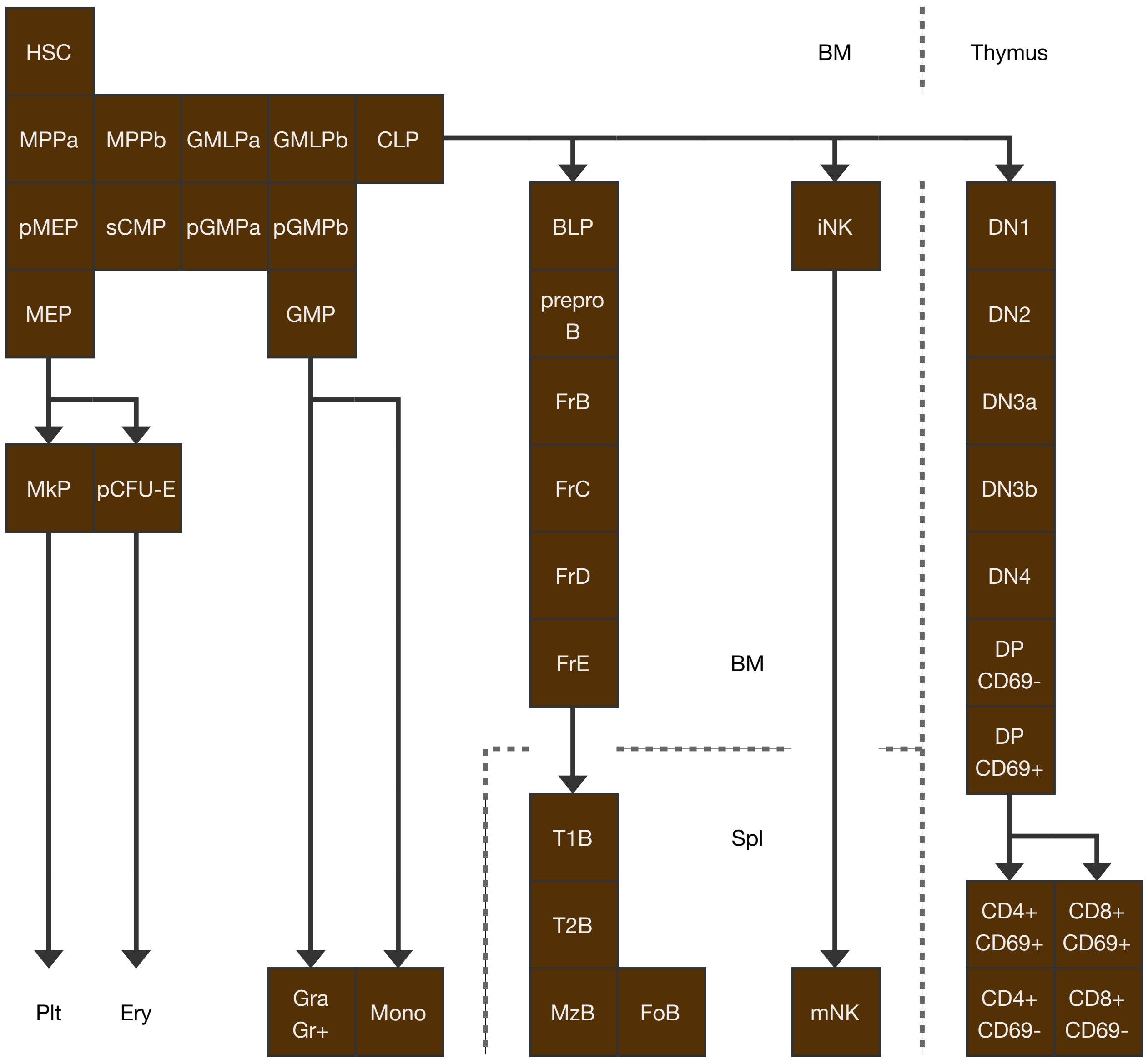

Supplement: S5 Fig — The schematic diagrams in panel (A-D) display Gene Expression Commons generated representation of expression patterns for transcription factors or genes annotated to transcription factor binding in S2 Table (A-C) and S4 (D). Gene lists were uploaded as csv files and analysis was performed using the Gene-set activity function. Expression levels are indicated with heatmaps reaching from dark Blue (Low-expression) to dark Brown (High-expression) as indicated. (PDF) [file pgen.1008280.s015.pdf]
