# Supplementary material for: PAX5 is part of a functional transcription factor network targeted in lymphoid leukemia
Source: PLoS Genet. 2019 Aug 5;15(8):e1008280. doi: 10.1371/journal.pgen.1008280 (PMC6695195; doi:10.1371/journal.pgen.1008280)
Supplement: S6 Fig — Peak files from ChIP-seq data of PAX5, EBF1, IKZF1 and RUNX1 in NALM6 cells (Fig 4A) were analyzed for motif enrichment using findMotifsGenome.pl in Homer (hg19 -size 200). Rank, enriched motif, P-value, % of target (T) and background (Bg) and best match to known motifs of Top 3 motifs plus PAX5, EBF1 and RUNX1 motif when present for each peak set are listed. (PDF) [file pgen.1008280.s016.pdf]

Figure S6

	Rank	*De novo* Motif	P-value	% T/Bg	Best match
NALM6 PAX5 peaks	1.	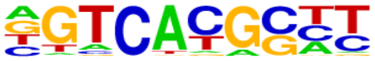	1e-886	31.6 / 10.9	PAX2
	2.	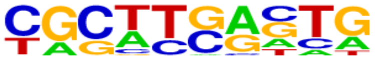	1e-784	29.7 / 10.5	PAX6
	3.	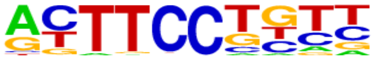	1e-662	35.1 / 15.4	ETV2 (ETS)
	5.	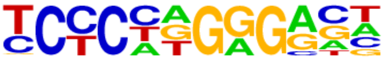	1e-480	13.9 / 3.8	EBF
	6.	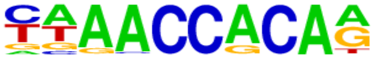	1e-368	11.6 / 3.4	RUNX
NALM6 EBF1 peaks	1.	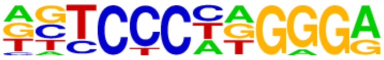	1e-8225	68.3 / 8.0	EBF
	2.	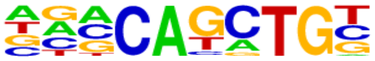	1e-677	36.3 / 18.1	PTF1a (bHLH)
	3.	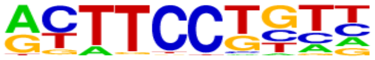	1e-666	37.1 / 18.8	ETV2 (ETS)
	4.	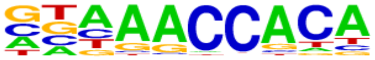	1e-411	17.7 / 7.5	RUNX1
NALM6 IKZF11 peaks	1.	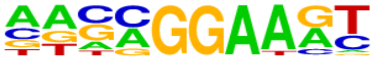	1e-3034	53.4 / 21.5	FLI1 (ETS)
	2.	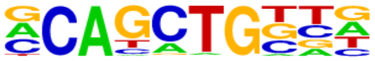	1e-1860	37.7 / 15.2	ASCL1 (bHLH)
	3.	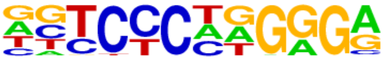	1e-1560	19.3 / 5.1	EBF
	4.	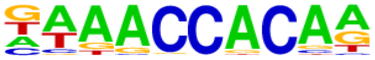	1e-1271	21.1 / 7.0	RUNX1
	9.	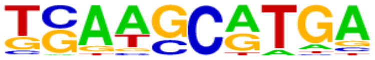	1e-187	7.7 / 3.9	PAX6
NALM6 RUNX1 peaks	1.	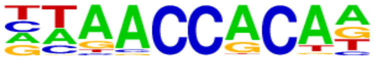	1e-2009	35.8 / 9.3	RUNX
	2.	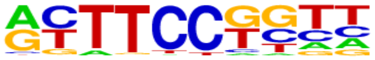	1e-1977	48.8 / 17.5	FLI1 (ETS)
	3.	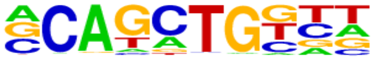	1e-1676	42.0 / 14.7	PTF1a (bHLH)
	4.	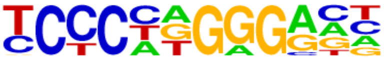	1e-1227	21.5 / 5.1	EBF
	11.	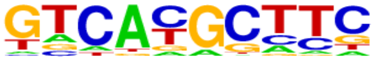	1e-127	6.9 / 3.2	PAX2
